# Effect of Collaborative Care on Persistent Postconcussive Symptoms in Adolescents: A Randomized Clinical Trial

**DOI:** 10.1001/jamanetworkopen.2021.0207

**Published:** 2021-02-26

**Authors:** Carolyn A. McCarty, Douglas F. Zatzick, Lyscha A. Marcynyszyn, Jin Wang, Robert Hilt, Thomas Jinguji, Celeste Quitiquit, Sara P. D. Chrisman, Frederick P. Rivara

**Affiliations:** 1 Seattle Children’s Research Institute, Center for Child Health, Behavior and Development, University of Washington, Seattle; 2 Department of Pediatrics, University of Washington, Seattle; 3 Department of Psychiatry and Behavioral Sciences, University of Washington, Seattle; 4 Harborview Injury Prevention and Research Center, University of Washington, Seattle; 5 Seattle Children’s Department of Psychiatry and Behavioral Medicine, University of Washington, Seattle; 6 Department of Orthopedics and Sports Medicine, University of Washington, Seattle

## Abstract

**Importance:**

Despite the high level of impairment for adolescents with persistent postconcussive symptoms, few studies have tested whether such problems can be remediated.

**Objective:**

To examine whether collaborative care treatment is associated with improvements in postconcussive, quality of life, anxiety, and depressive symptoms over 1 year, compared with usual care.

**Design, Setting, and Participants:**

The Collaborative Care Model for Treatment of Persistent Symptoms After Concussion Among Youth II Trial was a randomized clinical trial conducted from March 2017 to May 2020 with follow-up assessments at 3, 6, and 12 months. Participants were recruited from pediatric primary care, sports medicine, neurology, and rehabilitation clinics in western Washington. Adolescents aged 11 to 18 years with a diagnosed sports-related or recreational-related concussion within the past 9 months and with at least 3 symptoms persisting at least 1 month after injury were eligible. Data analysis was performed from June to September 2020.

**Interventions:**

The collaborative care intervention included cognitive behavioral therapy and care management, delivered mostly through telehealth, throughout the 6-month treatment period, with enhanced medication consultation when warranted. The comparator group was usual care provided in specialty clinics.

**Main Outcomes and Measures:**

Primary outcomes were adolescents’ reports of postconcussive, quality of life, anxiety, and depressive symptoms. Secondary outcomes were parent-reported symptoms.

**Results:**

Of the 390 eligible adolescents, 201 (51.5%) agreed to participate, and 200 were enrolled (mean [SD] age, 14.7 [1.7] years; 124 girls [62.0%]), with 96% to 98% 3- to 12-month retention. Ninety-nine participants were randomized to usual care, and 101 were randomized to collaborative care. Adolescents who received collaborative care reported significant improvements in Health Behavior Inventory scores compared with usual care at 3 months (3.4 point decrease; 95% CI, −6.6 to −0.1 point decrease) and 12 months (4.1 point decrease; 95% CI, −7.7 to −0.4 point decrease). In addition, youth-reported Pediatric Quality of Life Inventory scores at 12 months improved by a mean of 4.7 points (95% CI, 0.05 to 9.3 points) in the intervention group compared with the control group. No differences emerged by group over time for adolescent depressive or anxiety symptoms or for parent-reported outcomes.

**Conclusions and Relevance:**

Although both groups improved over time, youth receiving the collaborative care intervention had fewer symptoms and better quality of life over 1 year. Intervention delivery through telehealth broadens the reach of this treatment.

**Trial Registration:**

ClinicalTrials.gov Identifier: NCT03034720

## Key Points

**Question:**

What is the effectiveness of collaborative care with cognitive behavioral therapy for treating adolescents with persistent postconcussive symptoms?

**Findings:**

In this randomized clinical trial of 200 adolescents, those who received collaborative care reported fewer postconcussive symptoms at 3 and 12 months and higher health-related quality of life at 12 months compared with a control group receiving usual care.

**Meaning:**

These findings suggest that collaborative care with cognitive behavioral therapy is a promising treatment to alleviate symptoms and improve functioning for adolescents with persistent postconcussive symptoms.

## Teaser

This randomized clinical trial examines whether collaborative care treatment for adolescents with persistent postconcussive symptoms is associated with improvements in postconcussive, quality of life, anxiety, and depressive symptoms over 1 year, compared with usual care.

## Introduction

Sports-related concussions account for nearly 15% of all injuries in high school athletes.^1^ Although most individuals experience symptom recovery within 30 days, a subset of 20% to 30% of patients report symptoms that extend longer,^2^ clinically characterized as persistent postconcussive symptoms (PPCS). The most common symptom complaints with PPCS include fatigue, worsening emotional issues (eg, depression, irritability, and anxiety), poor concentration and memory, and headaches.^3,4^ Adolescents with PPCS experience a number of disruptions in their daily routine that can decrease their quality of life,^5,6^ often lasting for many months and even up to a year.^7,8^

Despite the substantial clinical need among pediatric patients with PPCS, there is a lack of high-quality evidence guiding best treatment practices.^4,9^ Psychoeducation about concussion for parents and children is commonly given but has been found to be insufficient in improving outcomes.^10^ Cognitive behavioral therapy (CBT) techniques, such as teaching patients how to cope with and manage symptoms or reframing negative thinking around symptoms, have been posited to play an important role in addressing the genesis and perpetuation of PPCS.^11^ Studies with adults suggest that CBT interventions that include these elements may relieve symptoms after traumatic brain injury.^12,13,14^ Yet, the literature is more scant for pediatric populations. One pre-post pilot study of CBT for adolescents with PPCS found that a brief 4-session treatment delivered weekly was associated with decreases in symptoms and improvements in quality of life.^15^

We have previously described how collaborative care health care delivery can be applied to PPCS management and treatment, by combining CBT with care coordination and enhanced medication consultation to address lingering symptoms when warranted.^16^ In our prior pilot trial^17^ testing this collaborative care model among adolescents with PPCS, we found that patients in both collaborative care and usual care groups showed symptom reductions in the first 3 months, but only those who received collaborative care demonstrated sustained improvements in PPCS and quality of life through 6 months of follow-up. Although the results were promising, they relied on an in-person care model that required substantial effort by families to access treatment.

The current investigation builds on the results of our prior pilot investigation.^18^ We modified the pilot intervention to allow for delivery over a secure synchronous videoconferencing platform, if preferred, and recruited patients from multiple clinics over a broader geographic region. In addition, we extended the study time frame to 1 year to allow for follow-up throughout the period for which functional impairment occurs in this population.

In this randomized clinical trial, we tested the hypothesis that this collaborative care treatment model would lead to improvements in postconcussive, anxiety, and depressive symptoms and quality of life over the course of 1 year, compared with usual care. We also examined other outcomes, including satisfaction, sleep quality, and headache pain, to broadly understand the impact on a range of symptoms experienced by adolescents with PPCS.

## Methods

### Study Population

Adolescents were recruited from pediatric primary care, sports medicine, pediatric neurology, and rehabilitation clinics in western Washington between March 2017 and May 2019, with follow-up through May 2020. Adolescents were eligible to participate if they were aged 11 to 18 years, had a medically diagnosed sports-related or recreational-related concussion within the past 9 months, and had at least 3 PPCSs persisting at least 1 month after injury. These criteria are aligned with the *International Statistical Classification of Diseases and Related Health Problems, Tenth Revision (ICD-10)* definition of postconcussion syndrome^19^ and have been used in other research studies of PPCS.^20^

Informed consent and assent were obtained before data collection, and study procedures were approved by the Seattle Children’s institutional review board (see Supplement 1 for the study protocol). An independent Data Safety Monitoring Board convened semiannually to review all possible adverse events and study recruitment. This study follows the Consolidated Standards of Reporting Trials (CONSORT) reporting guideline.

Adolescents were excluded from the study if they had spinal cord or other severe injuries, had a diagnosis of schizophrenia or psychosis, or presented with active, acute suicidal ideation. Patients with prior concussion and/or other preexisting psychological disorders were considered eligible. Of 1870 screened adolescents, 1480 (79.1%) were excluded for not meeting the inclusion criteria and 189 (10.1%) declined to participate.

### Study Procedures

The study design was a randomized clinical trial with collaborative care and usual care treatment groups in a 1:1 ratio according to computer-generated random assignments led by the study biostatistician (J.W.). After randomization, a letter and email were sent to the referring practitioner and the participant family to notify them about group assignment. One adolescent was excluded after recruitment because written informed consent could not be obtained. Data were collected using online surveys at baseline and again at 3-, 6-, and 12-month follow-up, facilitated by research associates who were blinded to collaborative care vs usual care group assignment.

### Collaborative Care Intervention

Adolescents randomized to the intervention received CBT, care management, and enhanced medication consultation, if warranted, throughout the 6-month treatment period. A flexible modular CBT format was used, in which the care manager could address any of the following topics according to the adolescent’s presenting concerns: pain management, relaxation and breathing, sleep hygiene, emotion regulation, mindfulness, challenging negative thinking, problem solving, crisis management, behavioral activation, avoidance, family communication, and/or the parent-child interaction.^16^ The adolescents were taught coping skills, relaxation, and cognitive strategies to manage their symptoms, while they were encouraged to pace their activities and increase activation as possible. We hoped to provide a minimum of 4 CBT sessions to all adolescents, with additional sessions if symptoms were not improving or more support was requested. CBT was primarily delivered in individual sessions with adolescents, although parental involvement was encouraged. Care management was most often conducted with the parent and addressed care coordination regarding medical treatment, social services, parent support, school advocacy, mental health, and other adolescent and parent concerns. Components of the intervention were offered in person or by video telehealth (see the study protocol in Supplement 1 for more details).^16^

Intervention patients were provided access to collaborative care for the duration of their symptoms, and access was terminated on symptom resolution or at the end of the 6-month intervention period. Symptom resolution was defined as either a 50% reduction in symptoms or reaching a subclinical threshold on measures of postconcussive, depressive, and anxiety symptoms. All care management and CBT were delivered by 1 of 2 study care managers, who were master’s level trained mental health professionals. Care managers were provided with weekly supervision with a supervising psychologist (C.A.M.) to discuss and review the modular CBT delivery. In addition, case conferences were convened weekly to discuss patients randomized to the intervention condition, attended by the care managers, a supervising psychologist (C.A.M.), a pediatrician (F.P.R.), a supervising psychiatrist skilled in the postinjury collaborative care model (D.F.Z.), and a psychiatric expert in pediatric psychopharmacology (R.H.). Care managers documented the amount of time and number of sessions delivered, CBT modules delivered, and involved parties in an online RedCAP database.

### Usual Care Control

Usual care was selected as the comparator condition, representing care being delivered to address PPCS as it naturalistically occurs in medical settings. Most adolescents in this study were receiving care from concussion specialists (sports medicine, rehabilitation medicine, and neurology) because we recruited more intensively in those settings, although some were receiving care from primary care practitioners. Individuals remained under the care of their chosen practitioner during the course of the study, and they received additional referrals as those practitioners deemed appropriate. Usual care was documented by obtaining parent report of health care utilization across settings over the course of the study.

### Primary Outcome Measures

#### Postconcussive Symptoms

The Health Behavior Inventory (HBI) is a 20-item questionnaire that assesses postconcussive symptoms on a 4-point scale, ranging from never to often, and yields total scores in cognitive and somatic domains (score range, 0-60 points, with higher scores indicating more symptoms). Both youth and parent report versions were administered. Both measures have demonstrated reliability and validity in youth with sports injury.^21,22^

#### Health-Related Quality of Life

The Pediatric Quality of Life Inventory (PedsQL) is a 23-item questionnaire that assesses physical, emotional, social, and school functioning (score range, 0-100, with higher scores indicating greater quality of life).^23^ The scale includes youth and parent report versions with demonstrated validity and reliability, including good-to-excellent internal consistency (Cronbach α = 0.88 for child and 0.90 for parent report).^24,25,26^ It has successfully been used in youth injury research,^27^ and a 4- to 5-point difference is clinically meaningful.^24^

#### Depressive Symptoms

The Patient Health Questionnaire–9 (PHQ-9), which includes 9 questions based on *Diagnostic and Statistical Manual of Mental Disorders* (Fourth Edition) major depression criteria, was administered to assess youth depressive symptoms (score range, 0-27, with higher scores indicating worse symptoms).^28^ It has been found to have high sensitivity and acceptable specificity for the diagnosis of major depression in adolescent populations.^29^

#### Anxiety Symptoms

The 15-item anxiety subscale of the Revised Child Anxiety and Depression Scale-Short Version^30,31^ assessed adolescent and parent reports on adolescent anxiety on a 4-point scale (score range, 0-45, with higher scores indicating worse symptoms). Adolescents also reported on their anxiety using the Generalized Anxiety Disorder-7 item scale (GAD-7)^32^ during the prior 2 weeks (score range, 0-21, with higher scores indicating worse symptoms).

### Other Outcome Measures

We also included several outcome measures that were not specified in the study protocol, to explore the impact of the intervention on a broader range of outcomes. These measures included satisfaction with care, sleep quality, headache pain, and suicidal ideation.

#### Satisfaction With Care

The Client Satisfaction Questionnaire was administered at 6 months to assess satisfaction with care and services received for concussive symptoms. This measure uses a 1 to 4 scale to assess satisfaction across 8 items related to care received (score range, 8-32, with higher scores indicating greater satisfaction).^33^ An example item from this measure is, “How would you rate the quality of care you have received?” with 1 being poor and 4 being excellent.

#### Sleep Quality

The 10-item version of the Adolescent Sleep Wake Scale, including the domains of going to bed, falling asleep and reinitiating sleep, and returning to wakefulness, was used as a measure of sleep quality.^34^ Higher scores (score range, 1-6) indicate better sleep quality.

#### Headache Pain

The Traumatic Brain Injury-Quality of Life Headache Pain^35^ is a 13-item measure that asks participants to assess how often they experience different issues associated with headache pain using a Likert Scale. Higher scaled scores indicate more pain-related issues (score range, 38.9-72.6).

#### Suicidal Ideation

Adolescents’ responses on the final item of the PHQ-9, which asks about “thoughts that you would be better off dead or thoughts of hurting yourself in some way,” were used to assess suicidal ideation over time. Responses to this item were dichotomized as any suicidal ideation vs none.

### Statistical Analysis

Power analyses suggested that our study would have 0.80 power to detect a significant (2-sided *P* < .05) group-by-time interaction with a between-group effect size comparable to those of other large-scale collaborative care interventions (Cohen *d* = 0.36),^36,37^ estimating a final sample size of 180. Descriptive statistics were tabulated, and random mixed-effects generalized regression models^38^ were used to test the hypothesis that adolescents randomized to collaborative care would demonstrate greater improvement than adolescents randomized to usual care for both adolescent and parent reports of postconcussive symptoms, anxiety, depression, and quality of life outcomes over 12 months. Before these longitudinal regression analyses, we examined baseline group differences, as well as missing follow-up assessment rates. No variables were found to be imbalanced at baseline, and 96% to 98% follow-up was achieved for parents and adolescents at the 3-, 6-, and 12-month follow-up time points.

All primary statistical analyses were conducted with the intent-to-treat sample. The continuous dependent variables were postconcussive, anxiety, and depressive symptoms and quality of life, sleep, and headache outcomes measured at 3-, 6-, and 12-month assessments. Satisfaction with care was measured at 6 months only. The dichotomous suicidal ideation, antidepressant medication, and health care utilization variables were also analyzed at 3, 6, and 12 months as other outcomes. Mixed-effect regression models were fit, including time categories, intervention group, and group-by-time interactions, Adjusted mean difference or adjusted relative risk (aRR) and 95% CIs were derived from the models. Effect sizes at each time point for the continuous outcomes were measured by Cohen *d*, calculated by adjusted mean difference between the 2 groups divided by a pooled SD, adjusting for gender, age, and time elapsed since the concussion injury event. SAS statistical software version 9.4 (SAS Institute) was used for all analyses. Data analysis was performed from June to September 2020.

## Results

**Figure 1.**
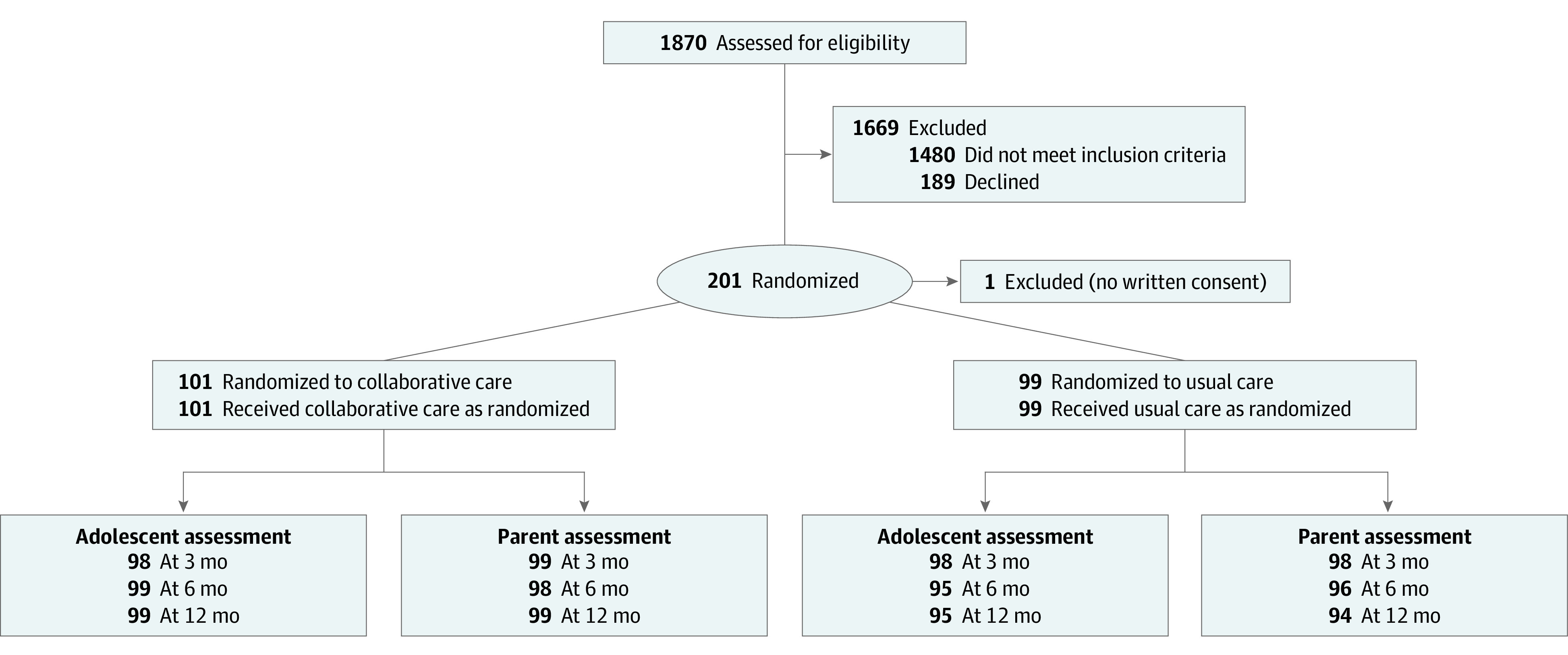
Flow of Participants Through the Randomized Clinical Trial

Figure 1 describes patient flow through the protocol. Of the 390 eligible adolescents, 201 (51.5%) agreed to participate, and 200 were enrolled (mean [SD] age, 14.7 [1.7] years; 124 girls [62.0%]); 99 participants were randomized to usual care, and 101 were randomized to collaborative care. Racial distribution was as follows: 164 participants (82.0%) were White, 17 (8.5%) were more than 1 race, 7 (3.5%) were Asian, 5 (2.5%) were Black, 3 (1.5%) were other, 1 (0.5%) was American Indian or Alaska Native, 1 (0.5%) was Native Hawaiian or other Pacific Islander, and 2 (1.0%) were of unknown race (Table 1). Seventeen participants (8.5%) identified as Hispanic or Latino ethnicity. More than one-half of the sample (103 participants [51.5%]) had a history of concussion, and a large proportion of participants indicated prior anxiety (62 participants [31.0%]), chronic headache (50 participants [25.0%]), and depression (36 participants [18.0%]). Nearly all participants were treated by specialty practitioners for their index clinic visit.

**Table 1.** Baseline Demographic and Clinical Characteristics

Characteristics	Patients, No. (%)		
	Total (N = 200)	Collaborative care (n = 101)	Usual care (n = 99)
Demographic			
Age, mean (SD), y	14.7 (1.7)	14.8 (1.7)	14.7 (1.7)
Female	124 (62.0)	60 (59.4)	64 (64.6)
Race			
White	164 (82.0)	82 (81.2)	82 (82.8)
>1 Race	17 (8.5)	8 (7.9)	9 (9.1)
Asian	7 (3.5)	2 (2.0)	5 (5.1)
Black	5 (2.5)	3 (3.0)	2 (2.0)
Other	3 (1.5)	2 (2.0)	1 (1.0)
American Indian or Alaska Native	1 (0.5)	1 (1.0)	0
Native Hawaiian or other Pacific Islander	1 (0.5)	1 (1.0)	0
Unknown	2 (1.0)	2 (2.0)	0
Hispanic or Latino ethnicity	17 (8.5)	9 (8.9)	8 (8.1)
Parent education			
High school graduate or less	13 (6.5)	7 (6.9)	6 (6.1)
Some college	35 (17.5)	15 (14.9)	20 (20.2)
Associate degree	32 (16.0)	13 (12.9)	19 (19.2)
Bachelor’s degree	73 (36.5)	39 (38.6)	34 (34.3)
Graduate school	46 (23.0)	26 (25.7)	20 (20.2)
Other	1 (0.5)	1 (1.0)	0
Annual household income, $			
<50 000	17 (8.5)	9 (8.9)	8 (8.1)
50 000-99 999	38 (19.0)	24 (23.8)	14 (14.2)
100 000-150 000	50 (25.5)	23 (22.8)	27 (27.3)
>150 000	83 (41.5)	39 (38.6)	44 (44.4)
Unknown	12 (6.0)	6 (5.9)	6 (6.1)
Clinical			
Prior diagnoses			
Learning disability	20 (10.0)	12 (11.9)	8 (8.1)
Attention deficit hyperactivity disorder	19 (9.5)	11 (10.9)	8 (8.1)
Anxiety	62 (31.0)	30 (29.7)	32 (32.3)
Depression	36 (18.0)	20 (19.8)	16 (16.2)
Prior diagnosed concussion	103 (51.5)	54 (53.5)	49 (49.5)
Chronic headache	50 (25.0)	23 (22.8)	27 (27.3)
Health care utilization 3 mo before index visit			
Mental health care	51 (25.5)	22 (21.8)	29 (29.3)
Primary care	161 (80.5)	80 (79.2)	81 (81.8)
Sports medicine	140 (70.0)	71 (70.3	69 (69.7))
Rehabilitation medicine	29 (14.5)	18 (17.8)	11 (11.1)
Other visit	85 (42.5)	41 (40.6)	44 (44.4)
Site of index visit for concussion care			
Sports medicine	176 (88.0)	87 (86.1)	89 (89.9)
Rehabilitation medicine	17 (8.5)	9 (8.9)	8 (8.1)
Neurology	3 (1.5)	2 (2.0)	1 (1.0)
Primary care practitioner	2 (1.0)	1 (1.0)	1 (1.0)
Other	2 (1.0)	2 (2.0)	0
Time since index injury, d			
0-30	6 (3.0)	2 (2.0)	4 (4.0)
31-60	117 (58.5)	62 (61.4)	55 (55.6)
61-90	36 (18.0)	19 (18.8)	17 (17.2)
91-120	21 (10.5)	8 (7.9)	13 (13.1)
121-180	10 (5.0)	5 (5.0)	5 (5.1)
>180	10 (5.0)	5 (5.0)	5 (5.1)

### Intervention Implementation

Of the 101 patients randomized to the intervention, all received some level of collaborative care treatment over 6 months. Overall, 61 adolescents (60.4%) received the intervention entirely by telehealth, 38 (37.6%) received a hybrid, and only 2 (2.0%) received the intervention entirely in person. The mean number of CBT sessions received was 8.4 (range, 2-22 sessions). In addition, families received a mean of 3.2 sessions of care management, with a total mean of 35 minutes of care management delivered to caregivers or adolescents, and 16 minutes spent with non–family members (eg, medical team or school personnel). Thirty-nine intervention patients (38.6%) received enhanced care in the form of medication consultation.

### Usual Care

Ninety-nine patients were randomized to the usual care group. Of the 98 who participated in the 3-month follow-up, 40 of their parents (40.8%) reported visits with a mental health professional over the first 3 study months (eTable 1 in the Supplement 2). In addition, 65 patients (66.3%) sought treatment from their primary care physician and 54 (55.1%) sought treatment from a sports medicine practitioner.

### Service Use and Antidepressant Medication

Comparisons for health service utilization between usual care and collaborative care are presented in eTable 1 in Supplement 2. Adolescents from the collaborative care group were more likely to receive mental health services (not including the study intervention) at 3 to 6 months, compared with usual care (aRR, 1.80; 95% CI, 1.13-2.85). There were no differences between groups in antidepressant medication use (eTable 2 in Supplement 2).

### Treatment Outcomes

**Figure 2.**
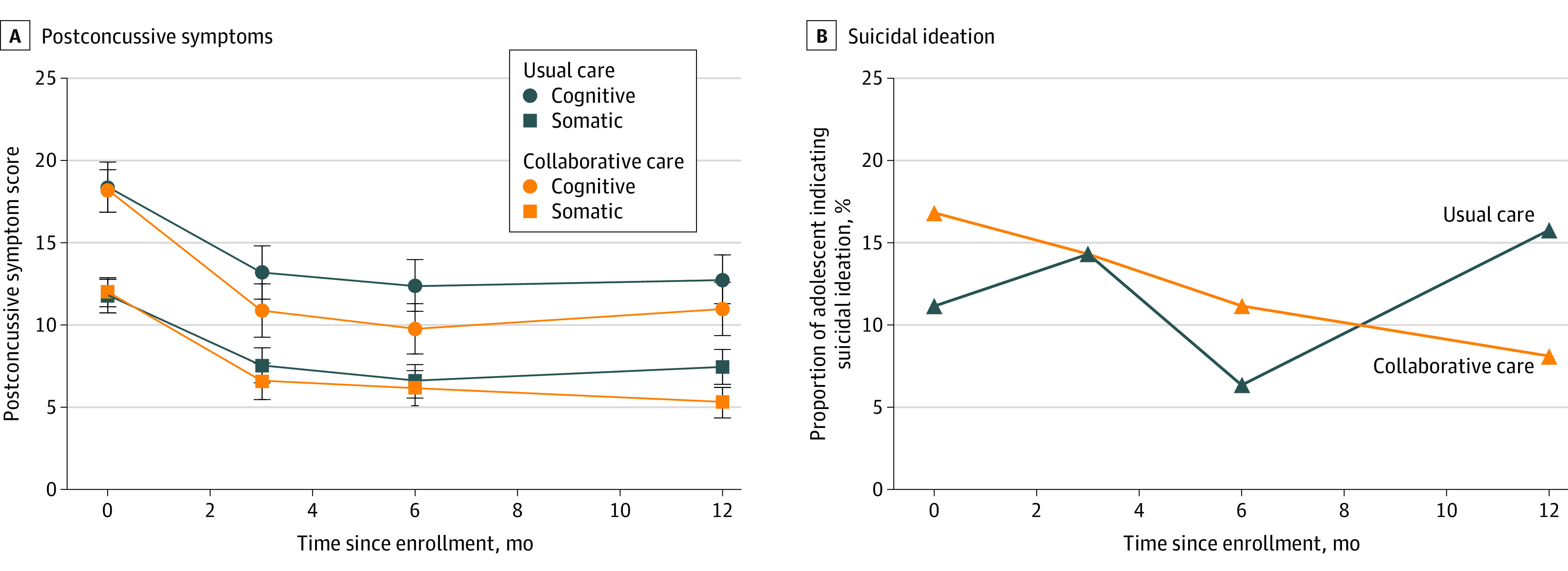
Postconcussive Symptoms and Suicidal Ideation in the Collaborative Care and Usual Care Groups by Adolescent Report Postconcussive symptoms (A) were assessed with the Health Behavior Inventory, a 20-item questionnaire that assesses postconcussive symptoms on a 4-point scale, ranging from never to often, and yields total scores in cognitive and somatic domains (score range, 0-60 points, with higher scores indicating more symptoms). Suicidal ideation (B) was assessed according to participants’ response to the final item on the Patient Health Questionnaire–9. Responses to this item were dichotomized as any suicidal ideation vs none.

**Figure 3.**
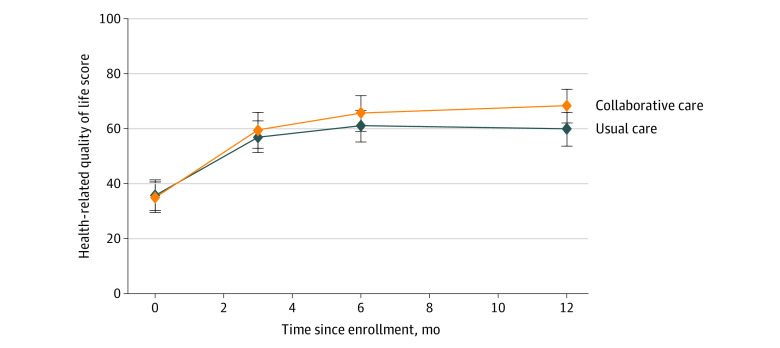
Health-Related Quality of Life Scores in the Collaborative Care and Usual Care Groups by Adolescent Report Health-related quality of life was assessed with the Pediatric Quality of Life Inventory, a 23-item questionnaire that assesses physical, emotional, social, and school functioning (score range, 0-100, with higher scores indicating greater quality of life).

Adolescents who received collaborative care had improvements in postconcussive symptoms compared with those who received usual care at 3 months and 12 months, according to youth report (Table 2), with effect sizes (Cohen *d*) of 0.26 and 0.32, respectively. Adolescents who received collaborative care reported significant improvements in HBI scores compared with usual care at 3 months (3.4 point decrease; 95% CI, −6.6 to −0.1 point decrease) and 12 months (4.1 point decrease; 95% CI, −7.7 to −0.4 point decrease). Figure 2 shows subscale differences on the HBI by group and time, with significant improvements in cognitive symptoms at 3 and 6 months for the intervention compared with usual care, and significant improvements in somatic symptoms at 12 months favoring the intervention. No differences were detected for parent report (eFigure 1 in Supplement 2). Significant improvements in health-related quality of life were demonstrated by adolescent report (Figure 3) but not by parent report (eFigure 2 in Supplement 2). For functional status at 12 months, adolescent report on the PedsQL showed greater gains for the intervention group compared with usual care (mean, 16.8 vs 12.1 points; difference, 4.7 points; 95% CI, 0.05 to 9.3 points). Cohen *d* for the PedsQL at 12 months was 0.29. eTable 3 in Supplement 2 shows subscale means by group over time, indicating that youth-reported emotional functioning improved more in youth receiving collaborative care at 6 and 12 months and that social functioning improved more for this group at 12 months compared with usual care. No differences emerged by group over time for adolescent depressive or anxiety symptoms.

**Table 2.** Outcomes for Collaborative Care vs Usual Care Groups Over the 12 Study Months

Outcomes	Score range	Baseline score, mean (SD)		Net difference from baseline between collaborative care and usual care, mean (95% CI)^a^		
		Collaborative care (n = 101)	Usual care (n = 99)	3 mo	6 mo	12 mo
Primary, adolescent report						
Postconcussive symptoms^b^	0 to 60	30.9 (11.1)	31.1 (11.8)	−3.4 (−6.6 to −0.1)^c^	−3.0 (−6.4 to 0.3)	−4.1 (−7.7 to −0.4)^c^
Quality of life^d^	0 to 100	66.5 (15.5)	66.6 (15.2)	1.7 (−2.3 to 5.6)	2.8 (−1.3 to 6.8)	4.7 (0.05 to 9.3)^c^
Depressive symptoms^e^	0 to 27	9.4 (5.8)	10.0 (5.8)	−0.3 (−1.8 to 1.1)	−0.2 (−1.6 to 1.2)	−1.1 (−2.7 to 0.5)
Anxiety symptoms^f^	0 to 21	6.8 (4.8)	7.7 (5.4)	−0.6 (−1.8 to 0.6)	−0.7 (−2.0 to 0.5)	−0.8 (−2.3 to 0.7)
Anxiety symptoms^g^	0 to 45	8.2 (6.7)	8.6 (6.9)	−0.9 (−2.4 to 0.6)	−0.9 (−2.3 to 0.6)	−1.7 (−3.4 to 0.1)
Secondary, parent report						
Postconcussive symptoms^b^	0 to 60	27.1 (11.6)	28.2 (12.5)	1.8 (−1.1 to 4.8)	−1.3 (−4.5 to 1.9)	−0.7 (−4.0 to 2.5)
Quality of life^d^	0 to 100	65.7 (16.2)	63.8 (15.6)	−3.0 (−7.5 to 1.4)	−1.2 (−5.6 to 3.2	−1.5 (−6.1 to 3.1)
Depressive symptoms^e^	0 to 27	7.4 (5.0)	7.7 (4.9)	0.1 (−1.3 to 1.5)	0.2 (−1.2 to 1.6)	−0.05 (−1.5 to 1.4)
Anxiety symptoms^f^	0 to 45	4.7 (3.7)	5.7 (4.2)	0.3 (−0.8 to 1.3)	0.04 (−1.0 to 1.1)	0.4 (−0.7 to 1.6)
Other, adolescent report						
Sleep quality^h^	1 to 6	3.8 (0.8)	3.9 (0.9)	0.3 (0.2 to 0.5)^c^	0.2 (0.04 to 0.5)^c^	0.4 (0.2 to 0.6)^c^
Headache pain^i^	38.9 to 72.6	57.6 (5.0)	58.3 (5.0)	0.2 (−1.9 to 2.3)	−0.4 (−2.6 to 1.8)	−1.6 (−3.9 to 0.7)

^a^ Means were derived from mixed-effect models and are adjusted for gender, age, and time elapsed since the concussion injury event.

^b^ Determined by responses to the Health Behavior Inventory.

^c^ Differences between groups are statistically significant (*P* < .05).

^d^ Determined by responses to the Pediatric Quality of Life Inventory.

^e^ Determined by responses to the Patient Health Questionnaire–9.

^f^ Determined by responses to the Generalized Anxiety Disorder 7-item Scale.

^g^ Determined by responses to the Revised Child Anxiety and Depression Scale-Short Version (anxiety subscale).

^h^ Determined by responses to the Adolescent Sleep Wake Scale.

^i^ Determined by responses to the Traumatic Brain Injury-Quality of Life Headache Pain.

For the other outcomes, we found between-group differences on 3 of 4 measures. Sleep quality showed improvement for youth receiving collaborative care at all time points, compared with usual care (Table 2) and for all sleep subscales at 3 and 12 months compared with usual care (eTable 4 in Supplement 2). There were no group differences across time for headache pain (Table 2). Adolescents who received collaborative care treatment and their parents reported higher levels of satisfaction with care, measured at 6 months corresponding to the end of the treatment phase, with a possible range of 8 (lowest satisfaction) to 32 (highest satisfaction). For adolescents in the intervention group, overall satisfaction score was a mean (SD) of 28.1 (3.6) compared with 26.4 (5.0) among usual care group participants (1.8 point increase; 95% CI, 0.5 to 3.0 points; *P* = .01). Parent satisfaction score was a mean of 28.6 (3.6) among those in the intervention group, compared with 26.4 (4.3) among those in the usual care group (2.1 point increase; 95% CI, 1.0 to 3.3 points; *P* < .001). The proportion of participants reporting suicidal ideation at the 12-month follow-up decreased significantly among adolescents receiving collaborative care compared with usual care (aRR, 0.33; 95% CI, 0.13-0.86) (eFigure 3 in Supplement 2).

During the 12-month study period, 3 youth in the collaborative care group and 4 in the usual care group required psychiatric hospitalization or residential care, and 3 in the collaborative care group and 2 in the usual care group had a suicide attempt. The independent Data Safety Monitoring Board did not believe these events were associated with study participation or the intervention.

## Discussion

This randomized clinical trial examined the effectiveness of a collaborative care intervention delivered largely remotely by master’s level care managers in addressing PPCS among adolescents. The overall pattern of findings suggest that although both groups improved over time, adolescents gained additional benefit from collaborative care, as evidenced by decreases in their reports of postconcussive symptoms and improved quality of life over the course of the following year. The improvement in health-related quality of life at 12 months was in the range of what is considered clinically meaningful according to other research.^24^ Results for other outcomes were mixed, with no evidence of differential change for anxiety, depressive, or headache pain symptoms, but decreased suicidal ideation and increased sleep quality were seen among intervention participants, although these 2 outcomes were not specified a priori and should be viewed as more exploratory. The lack of differential outcomes between groups for depressive and anxiety symptoms was surprising and contrary to our hypothesis, given the delivery of CBT with modules that include evidence-based practices to address such symptoms for intervention youth, such as behavioral activation, addressing avoidance, and cognitive restructuring. More research is needed to understand what treatment elements can help to ameliorate these symptoms.

Another study^15^ examined CBT specifically as a treatment for adolescent PPCS and found symptom reduction and quality of life increases 1 month after 2 to 5 sessions of treatment. In their protocol, participants received psychoeducation, activity scheduling, sleep training, relaxation training, and cognitive restructuring, elements that were similar to the CBT content delivered in our study, although we also offered additional modules, longer treatment, care management, and medication consultation when needed.

The current results are similar to our previous pilot trial of collaborative care (Collaborative Care Model for Treatment of Persistent Symptoms After Concussion Among Youth),^17^ although that study found differences both in adolescent and parent report on symptoms and functioning at 6 months. The current study was powered to detect changes in the primary outcomes, unlike the pilot study. Adolescent report may be a more appropriate indicator of PPCSs and functioning, because some of the indicators may not be directly observable by parents, particularly for the emotional items. Another analysis using this same sample of adolescents with PPCS indicated that reliability and correlation of adolescent vs parent report symptoms were modest overall and that adolescents tended to report both more symptoms and higher quality of life, aligned with previous studies examining parent-child agreement on psychosocial measures (A. J. Johnson, MD, C. A. McCarty, PhD, L. A. Marcynyszyn, PhD, D. F. Zatzick, MD, S. P. D. Chrisman, MD, MPH, F. P. Rivara, MD, MPH, unpublished data, January 2021). In the current study, changes were evident mostly at 1 year after enrollment, whereas the previous study only followed outcomes for 6 months. The current study differs from the original pilot trial in a few different aspects: CBT was delivered by the same master’s level interventionist who delivered care management, fewer care management sessions were delivered, and most of the treatment was delivered remotely. The telehealth delivery was a unique aspect that offered adolescents and families the opportunity to receive care at their own convenience without the typical barriers of transportation and more limited scheduling. More than 60% of the intervention sample received the full intervention remotely, and an additional 37.6% received at least 1 remote session. Both adolescents and parents were highly satisfied with collaborative care, more so than families who received usual care. Health care utilization was comparable between the collaborative and usual care groups with 1 exception: intervention participants were more likely to have a mental health visit 3 to 6 months after study entry, which may be associated with the active care management provided as part of the intervention.

### Limitations

This study has limitations that should be addressed. Because collaborative care was a bundled intervention, it is not possible to separate the specific components composing it, such as CBT, care management, and medication consultation. This type of intervention is meant to address the full gamut of needs with which patients with comorbid mental and physical health concerns may present, offering an individualized and a stepped care approach instead of dismantling particular treatment elements.^39^ In addition, because the adolescents participating were unblinded as to intervention status, there is a possibility of social desirability bias, and receiving more visits as part of the study intervention (CBT, care management) may have impacted their satisfaction. Furthermore, our study sample had a higher proportion of girls compared with boys and was predominantly White, with high socioeconomic status. Previous research has suggested disparities in adolescents who present for subspecialty concussion care, with non-Hispanic individuals and those with private insurance more likely to have health care visits. Thus, our sample may be representative of who presents for concussion care, but it also may be limited in generalizability to all who have sustained PPCS.^40^

## Conclusions

Overall, this study suggests that collaborative care with CBT may be helpful to adolescents experiencing PPCS for symptom reduction and improving sleep, functioning, and suicidal ideation. However, differential outcomes may not be evident at the start of collaborative care treatment, but appear to emerge more clearly over the course of a year. Importantly, collaborative care was not associated with depressive and anxiety symptoms above and beyond usual care, which was surprising. Providing collaborative care via remote delivery was convenient and accessible, making the intervention more generalizable, especially now that telehealth has been widely adopted.

## Notes

Supplement 1. **Trial Protocol**

Supplement 2. **eTable 1.** Proportion of Adolescents Using Healthcare Services in the Collaborative Care and Usual Care Groups Across Time **eTable 2.** Proportion of Adolescents Receiving SSRIs and Tricyclic Antidepressants in the Collaborative Care and Usual Care Groups Across Time **eTable 3.** Health Related Quality of Life Subscales in the Collaborative Care and Usual Care Groups Across Time, Mean (SD) **eTable 4.** Sleep Quality Differences in the Collaborative Care and Usual Care Groups Across Time, Mean (SD) **eFigure 1.** Post-Concussive Symptoms (HBI) in the Collaborative Care and Usual Care Groups by Parent Report **eFigure 2.** Health-Related Quality of Life (Peds-QL) in the Collaborative Care and Usual Care Groups by Parent Report **eFigure 3.** Proportion of Adolescents Indicating Suicidal Ideation in the Collaborative Care and Usual Care Groups Across Time

Supplement 3. **Data Sharing Statement**
