# Supplementary material for: Effect of Collaborative Care on Persistent Postconcussive Symptoms in Adolescents: A Randomized Clinical Trial
Source: JAMA Netw Open. 2021 Feb 26;4(2):e210207. doi: 10.1001/jamanetworkopen.2021.0207 (PMC7910815; doi:10.1001/jamanetworkopen.2021.0207)
Supplement: Supplement 1. — Trial Protocol [file jamanetwopen-e210207-s001.pdf]

PROTOCOL TITLE:

-Collaborative Care Model for Treatment of Persistent Symptoms After Concussion Among Youth (Collaborative Care Study)

PRINCIPAL INVESTIGATORS:
Frederick P. Rivara, MD, MPH
Center for Child Health, Behavior, and Development
206-744-9449
fpr@uw.edu

Cari McCarty, PhD
Center for Child Health, Behavior, and Development
206-884-8243
Cari.mccarty@seattlechildrens.org

Doug Zatzick, MD
University of Washington
206-744-6701
dzatzick@uw.edu

Table of Contents

1.0 Objectives 2
2.0 Background 3
3.0 Inclusion and Exclusion Criteria 6
4.0 Study-Wide Number of Subjects 7
5.0 Study-Wide Recruitment Methods 7
6.0 Multi-Site Research 7
7.0 Study Timelines 7
8.0 Study Endpoints 7
9.0 Procedures Involved 8
10.0 Data and Specimen Banking 16
11.0 Data Analysis/Management 16
12.0 Confidentiality 20
13.0 Provisions to Monitor the Data to Ensure the Safety of Subjects 21
14.0 Withdrawal of Subjects 21
15.0 Risks to Subjects 22
16.0 Potential Benefits to Subjects 25
17.0 Vulnerable Populations 25
18.0 Community-Based Participatory Research 26
19.0 Sharing of Results with Subjects 26
20.0 Setting 26
21.0 Resources Available 26

22.0 Prior Approvals 29
23.0 Recruitment Methods 29
24.0 Use of Social Media 31
25.0 Local Number of Subjects 31
26.0 Provisions to Protect the Privacy Interests of Subjects 31
27.0 Compensation for Research-Related Injury 32
28.0 Economic Burden to Subjects 32
29.0 Consent Process 32
30.0 Process to Document Consent in Writing 34
31.0 Drugs or Devices 34
32.0 Good Clinical Practice 34

1.0 Objectives

1.1 Specific Objectives:

Objective 1: To determine the effectiveness of a stepped-collaborative care intervention model in reducing post-concussive and co-occurring psychological symptoms in adolescents with persistent post-concussive symptoms after sports-related concussion.

Objective 2: To examine the effectiveness of the intervention in improving function and health-related quality of life amongst adolescents with persistent symptoms after sports-related concussion.

Objective 3: To explore differences in school performance between treatment groups.

Objective 4: To explore the heterogeneity of treatment effects in the primary and secondary outcomes by examining the interaction of the treatment effect with group membership in distinct subgroups of the population.

1.2 Hypotheses to be tested

Hypothesis 1: Adolescents receiving a collaborative care intervention will demonstrate clinically and statistically significant reductions in post-concussive symptoms, depressive and anxiety symptoms over the course of the 12-month study, compared to usual care control group.

Hypothesis 2: Adolescents who receive a collaborative care intervention will exhibit a clinically meaningful improvement in function and health-related quality life, compared to usual care control group.

Hypothesis 3: Adolescents who receive the collaborative care intervention will receive individualized treatment and community resource linkages which will improve their school performance, compared to usual care control group.

Hypothesis 4: Three distinct subgroups are expected to emerge from the study population: adolescents who recover from symptoms, adolescents with chronic psychosocial problems, and adolescents whose symptoms wax and wane over time. A greater proportion of adolescents who recover from symptoms will emerge in the treatment group, compared to controls.

2.0 Background

2.1 Relevant prior experience and gaps in current knowledge:

While post-concussive symptoms following sports-related concussion are typically transient and resolve spontaneously within two weeks of concussive injury, 14% or more of youth who sustain concussion experience significant morbidity that can persist well beyond the normal disease course.1 Furthermore, post-concussive symptoms commonly co-occur with affective symptoms including depression and anxiety which when present can prolong recovery from primary post-concussive symptoms.2,3 Together, persistent physical and psychological symptoms confer protracted functional impairment and create a significant burden for affected youth, their family, and school.2, 4 Currently, there are no evidence-based guidelines to inform treatment of persistent post-concussive symptoms in youth and adolescents.

2.2 Relevant preliminary data:

In response to the dearth of evidence-based treatment approaches for youth with persistent post-concussive symptoms, we developed a novel collaborative care treatment model that simultaneously targets post-concussive symptoms and co-occurring depression and anxiety. Adolescents and their family members receive patient navigator care management services that bridge post-injury care across acute care, specialist and primary care health service delivery sectors, in addition to cognitive behavioral psychotherapy. Patients who remain symptomatic after initial treatment efforts receive stepped-up care that may include psychopharmacologic consultation. We have demonstrated feasibility of the intervention model through a pilot randomized-control trial of 49 adolescents with persistent post-concussive symptoms recruited from a regional children's hospital.5 Participants assigned to the intervention condition demonstrated significant and clinically-meaningful reductions in post-concussive and depressive symptoms as well as health-related quality of life as compared to adolescents in the usual care arm of the trial. The proposed study will expand upon the pilot trial in order to further develop, implement, and test the impact of an innovative collaborative care approach tailored to the needs of a patient population who currently have no evidence-based options. We propose a broad reach intervention strategy that is designed to be readily implemented in acute, specialty and primary care medical settings. We will conduct a randomized comparative effectiveness trial with 200 youth, ages 11-18, suffering from ≥ 3 post-concussive symptoms at least 1 month after their sports-related injury. Adolescents will be randomized to collaborative care (intervention) or post-sports injury care as usual (control group) conditions.

2.3 Background for, rational for, and significance of the research:

Sports-related concussions are endemic amongst children and adolescents and constitute a major public health challenge, particularly when they fail to resolve in a timely manner. Estimates of sports-related concussion range from 1.6 to 3.8 million per year in the United States.6

While symptoms from sports-related concussion normally resolve spontaneously within days to weeks following injury, an estimated 14% or more of school-aged children experience significant morbidity that lasts for several months.1 Persistent post-concussive headache, fatigue, dizziness, and inattention confer marked functional impairment for affected youth and can significantly interfere with academic performance and social functioning.7-9

Affective symptoms, including depression and anxiety, commonly accompany post-concussive symptoms (PCS). One study estimated that 72% of child and adolescent patients with post-concussion symptoms reported at least 1 emotional symptom following their injury, and that many of them develop new psychiatric disorders following their concussion, most commonly depression or anxiety.10 Symptoms of depression correlate with other post-concussion symptoms,11 and may further prolong recovery from primary symptoms as has been demonstrated in patients with traumatic brain injury.2-4,10,12

Furthermore, recommended activity and school restrictions may contribute to increases in depression and anxiety of these previously healthy, active athletes.13 Taken together, the complexities of persistent post-concussive symptoms in conjunction with co-morbid psychological symptoms create a significant burden for the injured youth, their family, and school.

Currently, there is little evidence to guide screening, intervention and service delivery coordination for sports concussion exposed youth.14 Current clinical paradigms for chronic post-concussive symptoms emphasize education about symptoms, anticipatory guidance around gradual return to physical and cognitive activity, and reassurance of a full recovery.15 While these guidelines are adequate for patients with typical recovery, providers of patients who experience chronic symptoms are left with few treatment options because the evidence base for treatment of youth with persistent post-concussive symptoms is lacking.16 Moreover, there are no concrete guidelines for effective symptom management and no clinical intervention trials targeting persistent PCS in children and adolescents.16 The constellation of PCS is broad and encompasses multiple symptom domains, sometimes requiring attention from sub-specialists as well as coordination with schools.17 While physical symptoms are managed by primary care physicians and sub-specialists, psychological symptoms are common though often not addressed.10 Psychotherapy and medication management services can be difficult to access and are not incorporated into the standard of care.18 Thus, healthcare providers face a major challenge in managing patients with persistent post-concussive symptomatology.

Collaborative care has demonstrated effectiveness in treating chronic disorders where both physical and psychological symptoms are present, and may be well-suited to address issues of screening, intervention and services delivery coordination among sports injured youth. Collaborative care is a healthcare delivery model that integrates medical and mental health and allows for individuals to receive care that is titrated to align with their clinical needs. Given the constellation of somatic, cognitive, and emotional impairments associated with post-concussive symptoms, an integrated approach to symptom management which targets behavioral and physical symptoms in combination is a promising treatment paradigm.19-22

Large-scale randomized clinical trials, including our own work,23-25 have established the effectiveness of collaborative care interventions that combine care management, evidence-based pharmacotherapy, and CBT in treating pediatric and adult primary care patients with depressive, anxiety and post-traumatic stress disorders. Based on the burden of persistent symptoms and comorbidity, the effectiveness of collaborative care models, and the lack of existing interventions for persistent PCS, we have developed a collaborative care intervention with embedded cognitive-behavioral therapy, care management, and psychotropic medication consultation to address persistent PCS. Our pilot data show that this intervention had large, clinically meaningful effects on reducing persistent PCS and increasing quality of life for adolescents compared to usual care. Collaborative care interventions may be optimal for youth suffering from PCS presentations as these models can facilitate linkages across primary care pediatric, rehabilitation, specialty and school based service delivery sectors.

Cognitive-behavioral therapy (CBT) with or without combined pharmacological intervention is a robust evidenced-based treatment for psychiatric disorders including depression and anxiety; an emerging literature also supports CBT as an efficacious intervention for PCS.26,27

Cognitive-behavioral therapy has shown response rates of 60-80% among youth between ages 12-17 with depression and among youth between ages 7-17with anxiety disorders, with long-term outcomes equivalent to anti-depressant medication.28-30 Across injured and other trauma-exposed populations, CBT is the evidence-based treatment modality for anxiety most consistently recommended by best-practice treatment guidelines.31-34 CBT has also been successfully delivered by phone and video conferencing,35,36 and video conferencing treatment is commonly used by psychiatrists at SCH. Initial studies suggest that CBT intervention strategies can reduce persistent TBI symptoms, including memory impairment, difficulties with concentration, and somatic symptoms such as fatigue, sleep problems and irritability.37 An emerging evidence base suggests that pharmacological interventions may be an effective adjunctive tool in combination with psychotherapeutic interventions in the management of PCS, anxiety, and depressive symptoms in youth and adults.29,38-45 The Selective Serotonin Reuptake Inhibitor (SSRI) and Serotonin Norepinephrine Reuptake Inhibitor (SNRI) classes of anti-depressant medication

can be delivered safely to adolescents and young adults suffering from anxiety and depression with appropriate psychiatric oversight.38,46 Post-injury pharmacotherapy can target non-specific persistent TBI symptoms such as insomnia.46-48

A key translational research question is the extent to which collaborative care interventions with proven effectiveness can be broadly applied in real world primary and specialty care pediatric settings. Intervention models that bridge primary care, community and specialty care services in order to deliver evidence-based treatments are a crucial element of the integration of behavioral healthcare after sports-injury in youth.8 Epidemiologic data suggests that it may take years for trauma-exposed individuals with high mental health symptom levels and associated functional impairment to enter treatment.49 Effective interventions delivered in real world, non-specialty mental health settings face the challenge of incorporating both patient-centered supportive care and evidence-based treatment targeting post-concussive, depressive and anxiety symptoms.8,33 Intervention models that serve to link acutely exposed youth to evidence-based treatments have not been widely implemented and represent a crucial next step in adolescent sports-injury intervention development.8 Our initial studies show promise for the translation of collaborative care interventions across settings.

Summary: Youth who experience persistent PCS often suffer from prolonged symptoms, including depression and anxiety; these symptoms may negatively impact physical functioning, academic performance and overall quality of life for injured youth and their families. There is a dearth of evidence to guide screening, intervention and care coordination in this area, with no large scale randomized effectiveness trials evaluating treatments to address this issue among pediatric populations. We have developed and pilot tested a collaborative care intervention that encompasses care management, cognitive behavioral therapy, and psycho-pharmacology, when needed. In this application, we propose to rigorously test our intervention using a health systems framework, and to examine effects on symptoms, functioning, and academic outcomes over one year.

3.0 Inclusion and Exclusion Criteria

3.1 Eligibility Screening:

Potential adolescent subjects and their parents will be independently assessed by a research assistant or study coordinator to determine whether or not they meet eligibility criteria. Each adolescent and parent will be asked to complete the Health Behavior Inventory (HBI)[50] to determine current number and severity of symptoms (one HBI for past week and one HBI for prior to injury). Each adolescent and parent will also be asked to retrospectively describe the injury event in which the index concussion occurred. Adolescents with ≥ 3 HBI symptoms which have lasted for at least 1-month but less than 9 months and who meet inclusion criteria described in the next section of the protocol will be eligible for the study.

3.2 Criteria that will define who will be included or excluded in the study:

Inclusion: Male and female sports-injured adolescents ages 11-18 with a health care provider diagnosed concussion and with ≥ 3 HBI symptoms that have endured or worsened for at least 1-month but less than 9 months since injury will be included in the investigation if they and a parent can read and speak in English. The diagnosis of concussion will be made by a qualified medical provider. Adolescents with prior concussions will not be excluded. To enhance reach / breadth of applicability, all adolescents with a prior or current diagnosis and/or treatment for a mental, emotional or behavioral health disorder will be included in the study.51

Exclusion: Adolescents who require immediate intervention (e.g., acute suicidal ideation) will be excluded and evaluated by Dr. Zatzick, co-PI and a psychiatrist with extensive experience with acute post-injury evaluations in children, adolescents, and adults. Parents of adolescents who report that their child has ever had a diagnosis of schizophrenia or psychosis will be excluded from the study. Parents of adolescents that report concerns about their child's ability to communicate may be excluded from the study pending consult with PIs. Adolescents who have suffered spinal cord or other severe injuries that prevent participation will be excluded from the study.

3.3 Special Populations

Adults unable to consent are not included in this research
Adolescents and parents who do not read and speak English will not be included
Individuals ages 11 to 17 who are not yet adults are included in this research
Infants are not included in this research
Wards of the state are not included in this research
Pregnant women are not included in this research
Prisoners are not included in this research

4.0 Study-Wide Number of Subjects

4.1 The research will include 200 adolescents 11-18 years of age and their parents.

5.0 Study-Wide Recruitment Methods N/A

6.0 Multi-Site Research N/A

7.0 Study Timelines

7.1 Describe:

- Individual subject's participation in the study will last 12 months after consent and randomization
- We anticipate it will take 24 months to enroll all study subjects
- The estimated date to complete this study (complete primary analyses) is February 2021.

8.0 Study Endpoints

- Subjects will participate in the study for a year with final data on primary and secondary outcomes collected at 12 months post randomization

- There are no primary or secondary safety endpoints for this research.

9.0 Procedures Involved

9.1 Study Design

This investigation is a randomized comparative effectiveness trial designed to assess whether sports-injured adolescents randomized to a stepped care protocol will demonstrate reduced post-concussive, anxiety, and depressive symptoms when compared to adolescents randomized to a usual care control condition. The investigation will also evaluate the impact of the stepped care intervention on youth function including academic outcomes.

Two hundred sports-injured male and female adolescents ages 11-18 with at least 3 post-concussive symptoms that persist for 1 month or more but less than 9 months after injury will be recruited from a variety of settings and randomized into the study. Intervention subjects will receive treatment from a care manager over the course of 6-months after randomization. Intervention team members will work collaboratively with primary care providers to link physical and mental health care longitudinally through outpatient follow-up and community rehabilitation. Intervention subjects and their families and collaborative team members will share information and deliberate treatment decisions with each other in order to develop an individually tailored treatment plan. Stepped, higher intensity care will be available for intervention subjects with recurrent symptoms. Stepped up care will include CBT booster sessions targeting post-concussive and related symptom comorbidity as well as psychopharmacologic assessment. Adolescent subjects in the control group will receive care as usual from their health care providers, a standard that is ethically acceptable.

Post-concussive, anxiety, and depressive symptoms, functional status as measured by health related quality of life, health service utilization, and academic attendance and performance will be assessed by blinded research assistants 3-, 6-, and 12-months after randomization for all intervention and control group adolescents and parents.

9.2 Research procedures

Recruitment: Subjects will be recruited from schools, primary care medical pediatric clinics, sports medicine specialty clinics, pediatric neurology clinics, and rehabilitation medicine clinics throughout the Seattle and Western Washington region.

There will be active recruitment by weekly scanning of the appointment lists for the Sports Medicine, Neurology, Neurosurgery and Rehabilitation Medicine clinics at Seattle Children's Hospital main campus and four satellite locations, and the Sports Medicine and Rehabilitation Medicine clinics at Harborview Medical Center. Providers seeing potentially eligible subjects will be contacted prior to patient visits and reminded to provide the patient with study information. The research team will follow-up with patients that express interest.

Recruitment materials (flyers & brochures) describing the study will also be distributed to school nurses, athletic trainers, and pediatric clinics throughout the Puget Sound area to share with patients. They will also be posted at community locations across the Puget Sound area, on local community internet message boards, on the Seattle Concussions Collaborative Webpage (seattleconcussions.org) and in waiting rooms within the offices of local medical providers. A shorter recruitment message will be used to reach potential participants via social media platforms such as Facebook and Instagram. All recruitment materials will direct parents and adolescents who are interested in participating to contact the study staff for more information. We will follow-up with potential subjects who express interest. Follow-up activities will be conducted by research assistants or study coordinator. The goal of the follow-up activities will be to describe the study to potential subjects, answer questions, screen for eligibility and arrange for a consent meeting (either by phone or in-person) for those that wish to participate.

The screening will consist of determining if potential subjects meet eligibility criteria, including number and severity of symptoms as reported on the HBI. This will be done prior to obtaining consent. We are requesting an alteration of HIPAA authorization for recruitment and screening procedures so we can complete eligibility screen prior to consent. If potential subjects meet eligibility criteria, a consent conference will then be arranged, and adolescents and their parents will be consented to participate in the study at that time. After consent has been obtained, but before randomization, we will confirm with their treating provider that the adolescent has had a concussion diagnosis.

Randomization: Randomization will occur in a 1:1 ratio according to a computer generated random assignment sequence prepared by the study biostatistician.[52] Once generated, intervention and control group assignments will be entered into password protected tracking system with access limited to study coordinator, care manager, and PIs.. Randomization will be conducted by the research coordinator. After randomization occurs a letter and email notifying the referring provider and participant family will be sent to notify them of which group the participant has been randomly assigned.

Usual Care for Control Subjects: Control subjects will receive usual care. Usual post-injury care includes the routine use of sports medicine, rehab medicine, primary care and emergency department services, as well as the occasional use of specialty mental health services. Usual care has been selected for controls as it remains the optimal comparator condition for policy guidelines. Usual care does include referral to specialty providers caring for adolescents with concussion as needed.

Collaborative Care for Intervention Subjects:

Care management engagement: Adolescents randomized to the intervention group will be telephoned by a care manager team member. In order to enhance initial treatment engagement and therapeutic alliance during this

initial session, the care manager will elicit and target for amelioration each adolescent's and family's unique constellation of concerns.[53,54] The care manager will share the study team's 24-hour phone number and encourage calls for spontaneous questions and concerns both from the intervention adolescent and parent participants. The care manager will also elicit treatment preferences and will schedule ongoing times to meet the adolescent during the initial days and weeks post randomization.

Care management meetings with subjects can occur in-person, or by HIPAA compliant video conferencing or phone call. In-person meetings will be held at clinical sites throughout the region, as convenient for families. During this time period, the care manager will elicit information required to formulate a comprehensive post-injury treatment plan. The adolescent's family members will be integrated into the treatment plan whenever feasible and acceptable to the adolescent. Initial discussions between the care manager and the adolescent and family members may also highlight any issues and concerns related to return to sport or academic activity. The care manager will present this plan during the weekly intervention team meeting.

As in the previous intervention protocols, standardized instruments will be used to monitor post-concussive symptoms, headache, sleep, anxiety, and depressive symptoms at regular post-injury intervals. The intervention includes a motivational interviewing element embedded within care management that targets both treatment engagement and high risk behaviors that threaten recurrent injury. The motivational interviewing intervention element consists of a graded sequence of clinical tasks, including: a) eliciting from adolescents their views of the importance of changing, and of their confidence in being able to change behaviors, b) giving adolescents personalized feedback, and c) clarifying the adolescent's behavior change goals and action plans. This motivational interviewing intervention component has been successfully developed and implemented by the Harborview group over the past decade in single and multisite pragmatic clinical trials

Cognitive Behavioral Therapy: The intervention includes modular CBT targeting post-concussive, anxiety and depressive symptoms. In this CBT treatment, the adolescent is taught coping skills, relaxation strategies, and cognitive strategies to manage their symptoms, while they are encouraged to increase appropriate activation, including pacing of activities.

The treatment manual is intentionally flexible to allow for tailoring to the unique presentation and needs of the adolescent, including how many sessions (4-12 sessions total) and what specific modules of content to deliver. Cognitive Behavioral Therapy sessions will be tailored to adolescent preferences and may occur either in-person or by HIPAA compliant video conferencing or phone call. The first session involves psychoeducation and goal setting to collaboratively develop an individualized intervention plan and behavioral targets with the adolescent and their parent.

Specific cognitive behavioral therapy intervention elements covered in subsequent modules may include: pain management, problem-solving, behavioral activation, cognitive reframing and reattribution of symptoms, relaxation and imagery, emotion regulation, family communication skills, parent training skills, exposure exercises, sleep hygiene, or brief trauma support.

For adolescents who demonstrate adequate cognitive behavioral therapy readiness/motivation, homework assignments are given.[48,55] Treatment is delivered primarily in individual sessions with adolescents, although parent involvement in the treatment is strongly encouraged, with the following goals: 1) educating parents, 2) creating common goals and a framework for reaching those goals, and 3) understanding the parental role in support and stress processes.

The team members delivering CBT will work closely with the care manager to communicate progress and needs, and to support the adolescent in recovery and resumption of normal activities and functioning. Symptoms that are elevated (depression, anxiety, post-concussive symptoms) are monitored using standardized instruments on a session by session basis to inform how treatment is working and whether to increase the intensity of CBT through booster sessions or consider a psychiatric consultation regarding medication initiation or changes.

All cognitive behavioral therapy treatment cases will be supervised by Dr. McCarty, a licensed clinical psychologist who developed the modular treatment. In addition, weekly collaborative care team meetings will be used for ongoing case management. Based on the pilot study it is anticipated that over 85% of adolescents will complete at least 4 session based treatments. Cognitive behavioral booster sessions will be available to adolescents with recalcitrant symptoms as part of the stepped care algorithm

Computerized decision-support tool facilitated collaborative care team supervision: The care manager will participate in a computerized decision-support tool facilitated weekly supervision sessions with Drs. Zatzick, Rivara and McCarty. The computerized decision-support tool supervision procedure has been implemented and refined in prior stepped collaborative care interventions conducted by the study team.[56,57] The web-based data management system for this study will be built in REDCap and accessible to intervention team members 24 hours per day. The care manager will enter all assessments and treatment contacts into web-based forms.

Because information is available in near real time, the system can be used by the intervention team supervisors to monitor and support fidelity to the standardized implementation of the intervention. Utilizing the computerized decision-support tool, the care managers will present cases to the supervisory team. The weekly one hour sessions will include supervision and coaching in concern elicitation, cognitive behavioral and motivational interviewing techniques. Supervisors will also problem solve care management issues that arise around health service linkages and return to sport and school discussions. After this team discussion,

the care manager will discuss treatment options with the adolescent and family and come to a shared decision about treatment planning. Stepped-up care treatment options will also be discussed at the weekly team meetings.

Stepped-up care: Standardized instruments will be used to monitor post-concussive, anxiety, and depressive symptoms at regular post-injury intervals. Adolescents who do not meet symptomatic criteria for post-concussive, anxiety, or depressive symptoms and/or demonstrate a 50% reduction in symptoms will be considered treatment responders.[46-48]

Adolescents who respond to an initial course of treatment will receive ongoing monthly telephone contacts by the care manager. Adolescents who are partial or non-responders (i.e., continue to meet symptomatic criteria for high post-concussive symptoms levels as evidenced by ≥ 3 symptoms on the HBI and /or have not demonstrated a 50% HBI symptom reduction), or have high anxiety or depressive symptom levels (e.g., PHQ-9 symptoms ≥ 10) will receive stepped-up care.

Stepped-up care entails either receiving a treatment not previously delivered or alterations of a current treatment plan. Adolescents who remain symptomatic will continue to receive stepped-up evidence-based care and/or relapse prevention telephone contacts over the course of 6-months post study entry. For those few adolescents who have recurrence of symptoms or remain symptomatic at 6 months post-study entry, we will provide CBT booster sessions.

Medication consultation: The stepped-up medication intervention arm aims to initiate and ensure adequate follow-up of psychopharmacological treatment targeting PCS including insomnia, as well as anxiety and depressive symptoms.

A series of placebo controlled, blinded, randomized clinical trials have established the SSRI and SNRI classes of anti-depressants as safe and efficacious in treating anxiety and depression for adults and youth.[29,33,34,38-45,58] Recent guidelines endorse these agents as the first line medication treatment for these symptomatic disturbance disorders after traumatic injury exposure.[33,34,58] Treatment trials targeting both anxiety and depressive disorders, as well as preliminary studies by the study team, suggest that these medications can be safely and effectively delivered to adolescent patients, with appropriate psychiatric psychopharmacological supervision.[29,46,59] Other trials suggest that medication may also be effective in treating sleep disturbances associated with persistent PCS.[46,48,60]

The stepped care medication consultation option will start with an 1-hour initial evaluation by the study care manager in collaboration with Drs. Zatzick and Dr. Hilt; As in previous collaborative care interventions by the study team,[5,61] initial discussions of post-concussive psychopharmacology will occur during weekly team meetings with Dr. Zatzick. Dr. Hilt will be brought in for consultation in more complicated medication discussions and/or stepped up psychopharmacologic care.

Evaluation topics include the adolescent's current injury status; current and past comorbid medical conditions; current primary care, sports medicine and rehabilitation service use; current medication use including analgesics and psychotropic medications; prior psychotropic medication use; and attitudes and beliefs surrounding medication treatment.[38,46,48] Recommendations for medications will be made to the adolescent's primary care physician and specialty care provider who is managing the concussion. Adolescents will be allowed to add medications targeting specific symptoms (e.g., insomnia) and also to switch medications. Medication follow-up visits with the prescribing physician will be scheduled by them in order to track treatment response and medication side effects.

Final contact & completion of active intervention: The objective of the final contact phase is to articulate a specific plan for ongoing care. The 6-month post-injury time-point marks the completion of team intervention activities. For adolescents who remain symptomatic and/or have had major difficulties with the transition to community care, a more intensive, individually tailored relapse prevention plan will be devised. The relapse prevention plan will identify potential stresses, problem solve around potential triggers for recurrences, identify external sources of support, and strengthen primary care and community linkages

Blinded follow-up assessments: For all adolescents enrolled in the trial, follow-up surveys will be completed online or by telephone at 3-, 6-, and 12-months after the traumatic injury. The research assistants conducting any telephone follow-up interviews will be blinded to participant study group.

9.3 Describe:

- Procedures to lessen the probability or magnitude of risk associated with participation in research procedures are described in section 15 of this protocol.
- No drugs or devices will be used in this research.
- The source records that will be used to collect data about subjects (e.g. surveys, scripts, and data collection forms) are attached to the Click smart form.

9.4 Data will be collected online or by phone using the following assessment instruments:

History of sports concussion: Adolescents and parents will provide a retrospective report of previous concussion events or concussion-like symptoms they experience. This assessment will include time of previous concussion events, mechanism of injury, and resulting onset and duration of symptoms. An additional set of questions regarding re-injury during the study time period will be asked in the 12-month survey.

Health Behavior Inventory (HBI): A 20-item questionnaire that assesses PCS on a 4-point scale, ranging from "never" to "often," and yields total scores in

cognitive and somatic domains. The scale includes youth-report and parent-report versions with established reliability and validity in youth with sports-injury.[62]

Patient Health Questionnaire (PHQ-9): A 9-item questionnaire that measures severity of depressive symptoms. Reliability and validity of the PHQ-9 have been established in pediatric populations with injury.[63] Parental self-report of depressive symptoms will also be measured using the PHQ-9

Anxiety measures (GAD-7 & RCADS): We will use the GAD-7[64] and the 15 anxiety items of the Revised Child Anxiety and Depression Scale (RCADS)[65] for adolescent report on anxiety and parent report on adolescent anxiety.

Pediatric Quality of Life Inventory (PedsQL). A 23-item questionnaire that assesses physical, emotional, social and school functioning. The scale includes youth-report and parent-report versions.[66,67]

Adolescent Sleep Wake Scale. The 10 item version of the ASWS, including domains of falling asleep, reinitiating sleep, and returning to wakefulness will be used as a measure of sleep quality.

The TBI-QOL- Headache Pain. This 13 item tool that asks participants to estimate how often they experience different issues associated with headaches and headache pain using Likert scale (1-5).

School Performance. We will ask adolescents and parents at study entry about the child's experience with school since the concussion injury to include number of days missed, any concerns about school performance, impact on grades, and whether school provides academic support to kids with concussion. This will be repeated at each of the follow-up assessments. Participants' 1 year pre- and post-injury school attendance and grades will also be obtained from school records as a measure of academic outcomes.[46] We will re-code grading across schools on a 4 point GPA as we have done in prior studies of TBI in youth. We will convert standardized tests into percentile score based on state norms.

Client Satisfaction Questionnaire. The 8 item CSQ will be used at 6 months to measure adolescent and parent satisfaction with services; it has excellent correlation with changes in symptom score.[70]

Exposure to Life Stress. For adolescent subjects we will use the UCLA Reaction Index (RI) Trauma History to collect information at baseline and the Life Events Checklist (LEC) to collection information at the 6 and 12 month time points. The UCLA RI asks whether or not the respondent has ever experienced 10 specific and different traumatic events. The LEC asks the respondent about whether or not specific life events occurred in the past 6 months, if the event was good or bad, and the level of effect the event had on the respondent's live. For parent participants we will use an adapted version of the questionnaire used in the National Comorbidity

Study - Trauma History at baseline. This will ask about prior abuse, assault, witnessing or experiencing an accident, disaster, life threating illness, death of loved ones. At 6 and 12 month time points we will use the Stressful Life Events Scale (LTLES) which asks whether or not parent has experienced specific events. Prior investigation by members of the investigative team suggests that greater parental cumulative trauma burden is associated with greater adolescent traumatic stress scores in the wake of subsequent trauma exposure. In this current investigation, the study team will again measure parental cumulative trauma burden in order to assess the potential for moderation of intervention effects.

Prior psychiatric history: We will collect information on prior psychiatric disorders based on our experience with prior studies on adolescents and adults. We will collect information on prior history of treatment for psychiatric disorder, use of psychotropic medications, and prior post-concussive symptoms.[72,73]

Demographic Characteristics. Parents will be asked to describe family demographic characteristics such as marital, ethno-cultural heritage and number of children. Caregiver(s)' occupations and combined family income will be also be obtained and used as a measure of family resources.

Health Service and Medication Utilization.[48,74] Parent report will be used to assess adolescents pre- and post-sports injury health service utilization. Parents will report on ED and outpatient pediatric and any specialty sports medicine care. Parents will also report on types of medication used by adolescent. The following table provides a summary of data collection time points:

Construct	Measure	Screen	Baseline	3-Mo	6-Mo	12-Mo
History of index sports-injury concussion and any prior TBI		P	P			P
Post concussive symptoms	HBI	A, P	A, P	A, P	A, P	A, P
Depressive symptoms	PHQ-9		A, P, PS	A, P, PS	A, P, PS	A, P, PS
Anxiety symptoms	GAD-7, RCADS		A, PS	A, PS	A, PS	A, PS
Quality of life	PedsQL		A, P	A, P	A, P	A, P
Exposure to Life Stress - Adolescent	PTSD RI Trauma History, at baseline; Life Events Checklist – Child Form at 6 and 12		A		A	A

	months					
Exposure to Life Stress - Parent	NCS-AS measure at baseline; LTLES at 6 and 12 months		PS		PS	PS
Sleep	ASWS		A	A	A	A
Headache	TBI-QOL – Headache Pain		A	A	A	A
School attendance and performance	Questionnaire and school records		P, SR	P	P	P, SR
Satisfaction with care	CSQ				A, PS	
Prior psychiatric history	Questionnaire		P			
Demographic characteristics	Questionnaire		PS			
Health Service and Medication Utilization	Questionnaire		P	P	P	P

A= Adolescent, P=Parent Report of Youth, PS=Parent Self-Report, SR = School Records

10.0 Data and Specimen Banking – N/A

11.0 Data Analysis/Management

11.1 Data analysis plan and statistical procedures

Descriptive statistics for demographic variables, symptom levels, and functional status will be tabulated by the project statistician. All scales will be scored and subscales described. This process will include examining the data for missing values, appropriate ranges and outliers, as well as construction of consort flow diagrams. All primary statistical analyses will be conducted with the intent-to-treat sample.

The primary purpose of the statistical analyses is to examine and compare trends in quality of life and post-concussive, anxiety, and depressive symptoms longitudinally between adolescents in the intervention and control conditions. The effect of major interest will be the time-by-treatment group interaction term. We hypothesize that intervention adolescents will demonstrate greater improvement than controls in both self-report and parent report of post-concussive, anxiety, and depressive symptomatic and quality of life outcomes over the course of the year of trial participation. Specifically, the Primary Outcome Measures include the following:

- *Post-concussive symptoms measured with the HBI [Time Frame: During the12 months after enrollment.]*
    - *The central hypothesis is that the two groups will have different patterns of HBI scores over time, with the intervention group showing significant reductions when compared to controls.*

- *Health related quality of life as measured with the PedsQL [Time Frame: During the 12 months after enrollment]*
  - *The hypothesis is that the two groups will have different patterns of PEDSQL scores over time, with the intervention group showing significant reductions when compared to controls.*
- *Depressive symptoms measured by the PHQ-9 [Time Frame: During the12 months after enrollment.]*
  - *The hypothesis is that the two groups will have different patterns of PHQ-9 scores over time, with the intervention group showing significant. reductions when compared to controls.*
- *Anxiety symptoms measured by the GAD-7 [Time Frame: During the 12 months after enrollment]*
  - *The hypothesis is that the two groups will have different patterns of GAD-7 scores over time, with the intervention group showing significant reductions when compared to controls.*
- *Anxiety symptoms measured by the RCADS [Time Frame: During the 12 months after enrollment]*
  - *The hypothesis is that the two groups will have different patterns of RCADS scores over time, with the intervention group showing significant reductions when compared to controls.*

We will use mixed random effects generalized regression models to test this hypothesis for continuous and discrete outcomes.[76-79] These models permit the inclusion of subjects with missing data and allow for individual varying slopes and intercepts over time. In addition, these models will allow use of covariates to model potential sources of non-response bias and time-dependent covariates. This model also allows the specification of random or fixed effects and the form of the serial correlation over time (if heterogeneity changes over time). Prior to these analyses, we will examine baseline group differences using the appropriate statistics for the distribution of the variable. Although randomization should ensure balance between the two groups, it is essential to control for known confounders in the design and analysis to prevent a biased assessment of the treatment effect.

Baseline injury, demographic or clinical variables found to be statistically significant in this analysis will also be included as covariates in the regression models. Some attrition is expected in the study samples. In prior randomized trials of injured adolescents, the investigative group has achieved 6-12-month follow-up completion rates ≥ 90%.[25,46]Estimates derived from these rates are incorporated into descriptions of subject flow and power analyses. Assumptions about the nature of missing data are crucial to the type of statistical analysis chosen.[80-82] Full information maximum likelihood estimates from mixed random effects generalized linear models adjust for data missing at random (MAR). MAR data depends upon previously observed variables_ .[83]

We will use statistical logistic models to determine which, if any, demographic or clinical characteristics, including treatment group membership, are predictive of subject attrition. Any factors observed to explain trends in missing data would be used as covariates in subsequent analyses. In past studies, no sources of consistent variation to explain missing data were found. Based on our low attrition rates and the lack of consistent variation in past investigations we believe that MAR is a reasonable assumption. However, we will perform a sensitivity analysis using non-MAR techniques. The analysis of missing data is an area of ongoing development, and the investigative group will incorporate the most contemporary approaches in the final analyses.[81,82]

Secondary Outcome Measures will include:

- *School performance as measured by GPA [Time Frame: During the 12 months after enrollment]*
- *Return to full activities at school as measured by the CLASS [Time Frame: During the 12 months after enrollment]*
    - *The secondary hypothesis is that the two groups will have different patterns on the CLASS over time, with the intervention group showing significant reductions when compared to controls*

We will test for changes in the GPA at 12 months compared to pre-injury in the intervention group participants compared to those in the control group. We will also examine differences in the scores on standardized tests in the same fashion, as well as examine for differences in time lost from school, including missing whole days as well as missing partial days. Secondary analyses will explore the associations with gender, type of sports injury, pre-injury history of psychiatric disorder or visits and cumulative lifetime trauma history and any observed treatment effects.

We will examine heterogeneity of treatment effects in the primary and secondary outcomes by examining the interaction of the treatment effect with group membership in distinct subgroups of the population. These will be exploratory analyses. Based on our clinical experience, we hypothesize at least three groups of patients: (1) children with mild post-concussive symptoms who, while the symptoms last longer than for most children, nevertheless do resolve relatively quickly; (2) a group of children with persistent PCS and prior history of substantial psycho-emotional distress; (3) a group of children who have symptoms that affect their quality of life, but which wax and wane over time. We will explore defining these groups (1) a priori using clinical indicators and symptoms, and (2) empirically using the study sample data, such as latent profile analysis or trajectory analysis. The analyses will define these groups and examine the heterogeneous effects of the intervention on the recovery profiles of these groups.

11.2 Power analysis and sample size:

Power analyses were conducted using PASS software to determine the appropriate number of participants for the study.[84-86] Based in part on prior investigation by the study team, assumptions including 4 assessment points (baseline, 3-month, 6-month, and 12-month), equal correlations over time of ρ = 0.7, two-tailed alpha = 0.05, and 10% 12-month attrition were used for all power analyses. We used data derived from our prior larger scale R01 collaborative care investigations to estimate symptomatic and quality of life treatment effects for power calculations;[48,87] data from the smaller scale CARE4PCS pilot was used to corroborate treatment effect estimates derived from these larger scale trials.[88,89] The study team conservatively estimated that the 12-month persistent concussive symptom treatment effect will be between d = 0.35-0.40, using preliminary data derived from prior study team larger scale R01 collaborative care investigations.[25,72]

To estimate the power to detect a group by time interaction effect on persistent concussive symptoms we used the primary continuous HBI total symptom score with baseline mean = 33.0, and common SD = 11.0. The investigation will require recruitment of 200 subjects (100 subjects in each group) in order to retain 180 subjects at the 12-month post-injury follow-up. With a final sample of 180 subjects, the power to detect a significant group-by-time interaction with a between-group effect size of d = 0.36 on the HBI somatic symptom scale is 0.80.

The study team also estimated the power to detect between-group differences in 12-month treatment response rates based on a response cutoff level of ≥3 symptoms on the HBI. If at the 12-month study endpoint, 44% of subjects in the intervention group vs. 65% of subjects in the control group continue to meet PCS symptomatic criteria of ≥ 3 on the HBI, the power is = 0.80. Data from the CARE4PCS collaborative care pilot corroborate these estimates. The CARE4PCS pilot demonstrated a 0.74 treatment effect on the 6 month symptoms measured by the HBI; with 180 subjects the power to detect a significant treatment group difference is ≥ 90%. In our prior R01 study, the 12-month post-injury treatment effects for the Medical Outcomes Study Short Form 12/36 Physical Components Summary were approximately 0.30.[72] In the current R01 proposal we used the PEDS-QL study entry baseline of 59.0 and common SD of 14.4. Anticipating an effect size of d = 0.37 with 90 subjects in each group, the power to detect a significant group by time interaction effect is 0.80. The CARE4PCS pilot demonstrated a 0.66 treatment effect on the HBI; with 180 subjects the power to detect a significant treatment group difference is ≥ 90%.

The investigative team has extensive experience recruiting and retaining in clinical trials youth from the spectrum of real world settings included in the current randomized clinical trial including soccer associations, acute and primary care pediatric settings and sports medicine clinics. Dr. Zatzick has completed both prospective cohort and randomized clinical trials that have successfully recruited and retained youth ages 10-19 presenting to both acute care and primary care pediatric settings.[46,90-94] Dr. McCarty is currently recruiting a sample of 300 adolescents and their parents from primary care settings for a randomized trial of a health screening and feedback tool. Dr. McCarty has been the PI on

intervention trials in the schools where she has screened >1000 youth for depression. Dr. Rivara has a wealth of experience recruiting from clinical sites for studies, and has established mechanisms to facilitate this process. Dr. Rivara has conducted a randomized clinical trial for the prevention of abusive head trauma with 2738 mothers recruited from prenatal classes, 3 maternity units and 11 private practices.[95] He is currently involved in another clinical trial for the prevention of child abuse which recruited 100 physicians and 500 parents from 30 practices in Western Washington (2 RO1 DA021307-06). His prior CDC funded study on TBI recruited 927 participants from 10 hospital EDs.[96] Group Health in Seattle has 80,000 enrolled youth and treats approximately 400 concussions annually (See letter of support).[97-101]

We have conducted a review of patients treated at Seattle Children's in 2014 for sports related concussions and found that there were 530 children 11-18 years of age treated during the one year period. Of these, 40% were seen for persistent symptoms. We plan on randomizing 200 youth for the study over a 24 month; there thus should be an adequate number of subjects to meet this goal.

11.3 Quality control procedures:

Training Research Assistants: During the start-up phase of the protocol, the research assistants will be trained to a reliable standard with the assessment procedures through: 1) attendance of evaluation training workshops, 2) group practice interviews, and 3) individual supervision with Drs. Zatzick and McCarty.

Training Care Management Team: During the start-up phase of the protocol, the intervention team members will be trained in the collaborative care intervention. This training will occur as a case-based clinical practicum supervised by Drs. Zatzick and McCarty.

Fidelity to Intervention Treatment Model: Adherence to elements of the treatment model including care management, pharmacotherapy consultation, and CBT elements delivered will be documented by the care manager. A composite intervention treatment fidelity checklist will be entered into the computer decision support tool by care managers on a monthly basis. As an additional quality check, all CBT sessions will be audio-recorded and a subset independently coded for adherence.

12.0 Confidentiality

12.1 **Data Security:** Data from the study will be derived from subject responses to interviews, questionnaires, and, for some, medical records. All data will be collected specifically for research purposes. Individually identifiable private information will be collected from research participants. To ensure subject confidentiality, all research materials will be kept in a locked file cabinet in a locked research office.

All medical record information and other electronic data will be stored on password-protected computers and encrypted. After data is collected, information which would identify the subjects will be removed and code numbers used

instead. A study code will be assigned to each subject. All subject data will be linked to the study codes in one master file that will be encrypted and stored on a secure laboratory study computer configured behind the departmental firewall. No presentation or publication arising from this research will use subject names or other information that would allow subjects to be identified

12.2 Data management:

- Data will be managed by study team using survey management software (e.g. REDCap)
- Survey and test data will either be stored in HIPAA compliant computers and servers or in locked file cabinets
- Data will be stored for 5 years after completion of the study.
- Only the study team will have access to the data.

13.0 Provisions to Monitor the Data to Ensure the Safety of Subjects

- An external, independent oversight board selected by the PIs will provide ongoing monitoring of the study for futility, data integrity, and safety.
- Prior to protocol initiation, the oversight board will review the procedures and plans for safety monitoring. The board will review the recruitment and retention of participants and will monitor the occurrence of adverse events. These events include deaths, suicide attempts, severe medication side effects, study dropout, psychiatric hospitalizations, and clinical deterioration defined as the development of new suicidal or homicidal behaviors. The board will also conduct reviews of study progression and data integrity to include assessments of attainment of study recruitment milestones, adequacy of follow-up, and threats to internal validity such as differential drop-out of subject in the intervention versus control conditions.
- The safety information to be reviewed by the oversight board will be collected by the study team using case report forms.
- After each encounter with a subject, the study team will determine if there is a safety event to report and, if so, will complete a case report form.
- Safety data will be reviewed by the oversight board. The Oversight board will consist of three members: 1) An expert on youth TBI and concussion (Dr. Keith Yeates), 2) A quantitative biostatistician (Dr. Wayland Howard), and 3) A pediatric bioethicist (Dr. Chris Feudtner); who serves as the Chair of this committee
- The oversight board will meet formally via teleconference every six months. Each year, the oversight board will produce a report that summarizes 1) All serious and unexpected adverse events, 2) The committee's opinion as to whether safety, confidentiality, and privacy have been adequately assured by the investigators, 3) A summary of progress towards recruitment and follow-up goals. The yearly summary will be forwarded to the PIs. The PIs will in turn forward the summary to the Seattle Children's Research Institute IRB.

14.0 Withdrawal of Subjects

14.1 We do not anticipate circumstances under which subjects will be withdrawn from the research without their consent.

14.2 Subjects who choose to withdraw from treatment, or from the study entirely, will be tracked and will be reported on subsequent IRB status updates. Reasons for withdrawal will be recorded whenever possible.

14.3 Subjects may withdraw from treatment at any time by notifying study personnel. Subjects who withdraw from treatment may still participate in study assessments. Subjects who wish to completely withdraw from the study may also do so at any time by contacting the study investigators. If subjects withdraw from treatment and/or study participation, they will be referred back to their standard care providers for ongoing follow-up.

15.0 Risks to Subjects

15.1 Potential risks to subjects include the following:

- Emotional distress: The baseline and follow-up interviews may produce discomfort or anxiety secondary to the discussion of trauma-related and emotionally laden topics. Questionnaire items assess sensitive topics, including suicidality, anxiety, and depressive symptomatic distress.
- Testing burden: Participants may experience inconvenience and invasion of privacy from the in-person and telephone interviews. With regard to participant time, the screening and consent procedure and initial study instrument assessment packet is anticipated to require 60 minutes to complete. The 3-, 6-, and 12-month follow-up assessments are anticipated to require 45-60 minutes each to complete. The total anticipated time required for interview participation is 4-5 hours over the course of the year post injury. All study measures described in the approach section of the grant proposal have been used by the investigative group in previous investigations.
- Confidentiality: Adolescents and their parents will be asked to answer potentially sensitive questions about depression, suicide, and anxiety after consent during the initial assessment set of surveys. The 3-, 6-, and 12-month follow-up surveys will also include sensitive questions related to anxiety, depression, and suicide.

15.2 Protection against risks include the following:

Recruitment and informed consent: The research assistant or study coordinator will give each parent a brief overview of the study and ask the potential parent participant whether or not they would be interested in hearing a more detailed description and learning if their child might be eligible.

If the potential parent participant wants to learn more, they will be asked to complete a brief eligibility screening tool. We are requesting an alteration of HIPAA authorization for recruitment and screening procedures. If the adolescent meets eligibility criteria and the parent still wants to learn more about the study, the research assistant or study coordinator will give a copy of the consent form to the parent and review the entire form with them, allowing participants to ask questions or voice concerns. The following general principles of informed consent will be outlined in the consent form: 1) voluntary participation; 2) the right to refuse or withdraw from the study at any point in time; 3) that participation,

refusal or withdrawal from the study would in no way impact the participants' ongoing surgical or mental healthcare; 4) a description of the risks and benefits of participation, including the statement that the study entails a risk that the subject may find some of the questions distressing; 5) the nature and extent of confidentiality; 6) identification of whom to contact for problems that may occur in the course of the research protocol; and 7) that the subject will receive a copy of the consent form.

The recruitment procedure will also include discussion of payment for study participation over the course of the year after the injury. Parent participants will also prospectively consent to the release of school based records. Parents will also be informed that they may elect to continue to receive intervention team services even if they or their children wish to drop from the study. The research team will keep the signed parent consent form with study records as a method of documenting the consent procedure. After parent consent the eligible adolescent will go through a similar study assent procedure.

Apart from the potential for distress, invasion of privacy, confidentiality, and inconvenience of screening, there is minimal risk to subjects from data collection procedures. Significant risks to the health of subjects are low, given that the intent of the study is to compare usual care with an enhancement of usual care that is expected to be effective and safe. Upon enrollment, parent and adolescent participants will be given a study wallet card with the study phone number. All subjects will be in contact by phone with the study research assistants, and intervention subjects will be in frequent contact with their study care manager. Participants will be asked to notify the study personnel if any difficulties arise.

Life threatening conditions and clinical deterioration: The research group has previously developed a protocol in which the development of any potential life-threatening conditions, including acute suicidality, detection or suspected child abuse, or any other life-threatening condition (e.g., severe medication side effects), requires research staff to immediately contact a member of the clinical supervisory team (Dr. Zatzick, Dr. McCarty, or Dr. Rivara) for further guidance and instruction. Dr. Zatzick is a practicing trauma center youth and adult consultation liaison psychiatrist. Dr. McCarty is a Washington State licensed psychologist, with extensive experience in evaluating depression and suicidal behavior among adolescents. Dr. Rivara is a senior pediatrician experienced in child abuse assessment. In regards to suicidality, the study coordinator will receive an alert from REDCap if a participant meets the following criteria after completing the PHQ-9 assessment:

- PHQ-9, item 9 = 1 AND item 9a = yes OR
- PHQ-9, item 9 is > or = 2

This criteria and alert have been established to help us identify participants with potential suicidality for further assessment to determine next steps to take. If the participant is assigned to the control arm of the study, the study coordinator will

contact the on call member of the clinical supervisory team who will then contact the participant and initiate the self-harm protocol to determine risk level along with next steps. If the participant is assigned to the intervention arm of the study, the study coordinator will contact the care manager assigned to the participant who will reach out to the participant. The care manager, having developed a relationship with the participant, will be well positioned to initiate the self-harm protocol to determine the participant's level of risk. Care Managers will immediately contact the on call member of the clinical supervisory team for further guidance and instruction in all cases where participants' risk is assessed to be moderate or high (considered acute suicidality).

All consent forms will specify that all information collected in the study will be confidential, except for the reporting of child abuse as required by law. In the case in which threat may be recurrent or ongoing, the principal investigator will work with clinical intervention team members to develop a tailored safety protocol. The safety protocol will incorporate the general principles of ensuring participant safety, protecting privacy, minimizing participant distress, and referrals for care and support. The safety protocol will be individually tailored to the unique circumstances of each individual patient.

Also, any adolescent or parent in the intervention or control condition who demonstrates substantial clinical deterioration will be evaluated by a member of the clinical supervisory team. This approach to protection against risk has been successfully implemented in the University of Washington IRB approved and DSMB monitored safety protocol in previous investigations (University of Washington IRB approval #24069 and Seattle Children's IRB approval #15152).

Emotional distress: The study team will employ multiple procedures to reduce the potential risk of study procedures incurring emotional distress among injured trauma patients. First, the clinical supervisory team will train research assistants conducting interviews and administering questionnaires in empathic listening and interviewing skills.

Basic principles to be discussed during these trainings include the gradual pacing of interviews, allowing participants to return to or defer items that are the source of major distress, and when to stop an interview and page a member of the clinical supervisory team. In cases when a participant experiences extreme distress in responding to questions, a member of the clinical supervisory team will immediately be paged by the research assistant conducting the interview to perform an in-depth clinical evaluation of the participant. A self-harm assessment protocol will be utilized to follow-up for all participants who indicate suicidal ideation.

Testing burden: All attempts will be made to conduct baseline and follow-up interviews at times that are convenient for participants. If subjects experience fatigue or distress during an interview they will be allowed to discontinue. In cases when a participant experiences extreme distress in responding to questions, the principal investigator will immediately be paged by the research

assistants conducting the interview to perform an in-depth clinical evaluation of the patient.

Adolescents and parents will be reimbursed for the time required to complete study recruitment and follow-up procedures. Sports injured adolescents and their parents represent a challenging population to engage and follow-up with. It is anticipated that the initial telephone screen will require less than 30 minutes of each adolescent and parent. It is anticipated that the initial visit during which informed consent will be obtained and initial interviews completed will require approximately 90 minutes. Adolescents and parents will be reimbursed $40 and $20 respectively for this assessment. Follow-up interviews will require between 30-60 minutes of each adolescent's and parent's time, and each adolescent and parent will be reimbursed $20 for completion of the 3- and 6-month follow-up evaluation and $40 and $20 for completion of the 12-month evaluation. Adolescents and parents will each receive a total of $120 and $80 respectively over the course of the investigation. Payments increase over the course of the year in order to optimize longitudinal follow-up. The amount of these payments approximates subject reimbursements previously approved by the Seattle Children's Research Institute IRB for single and multi-site investigations of injured trauma survivors.

Maintaining confidentiality: Clinical interviews, subjective ratings, audio-taped conversations, electronic medical records, clinical registry tool, and other data will be obtained during the study. These data will be gathered strictly for research purposes and only accessed by study staff. To insure subject confidentiality, all research interviews and other research materials will be kept in a locked file cabinet in a locked research office, and all computers used in the collection and storage of data will be password protected. After data is collected, information which would identify the subjects will be removed and code numbers used instead. A study code will be assigned to each subject.

Staff training and ongoing mentorship in ethical conduct of research: The principal investigators and other study key personnel have completed the human subjects' training. Within the first months of initiation of the proposed investigation, the research coordinator and assistants will complete a general human subjects/HIPAA training and a 2-hour trauma specific didactic training conducted by Dr. Zatzick.

16.0 Potential Benefits to Subjects

16.1 Potential benefits that individual subjects may experience from taking part in the research:

Subjects in the intervention group may directly experience benefits from the proposed investigation. These include reductions in anxiety and depressive and other post-concussive symptom levels, pain and somatic symptom amplification, and functional disability.

17.0 Vulnerable Populations

17.1 Safeguards to protect persons who have not attained the legal age for consent for procedures involved in the research:

- The study team will obtain permission from one parent and assent will be obtained from the adolescent subjects who are younger than 18 prior to randomization of the adolescent subject or data collection from adolescent subjects. For adolescent subjects age 18, the study team will obtain consent from the adolescent. Adequate time will be provided for parents and adolescents to ask questions about the study and make an informed decision as to whether or not they consent to participate.
- This research is minimal risk as it involves no drugs or devices and is a test of a behavioral approach to treating concussed youth that has shown benefit in a pilot study.
- We believe it is essential to better understand the best approach for treating persistent concussion symptoms in adolescents and this can only be carried out using a controlled trial.
- We also believe that adolescents who enroll in this trial will obtain a potential benefit as they may experience a decrease in their persistent concussive symptoms and improvement in function.

18.0 Community-Based Participatory Research – N/A

19.0 Sharing of Results with Subjects

19.1 No one other than the study team will have access to identifiable data. Requests from subject families for their individual results will be considered by the PIs on a case by case basis.

20.0 Setting

20.1 Locations where the research team will conduct the research:

- Potential subjects will be recruited from multiple locations to include schools, primary care medical pediatric clinics, sports medicine specialty clinics, pediatric neurology clinics, and rehabilitation medicine clinics throughout Seattle and Western Washington State. Potential subjects will also be recruited via online advertising.
- The study coordinator, research assistants, care managers, and two of the three investigators will be located at Seattle Children's Research Institute. Recruitment, eligibility screening, consent, and data collection procedures will be conducted via telephone, text, email, online advertising and survey, and in-person meetings; interventions will be done in person or by HIPAA-compliant video conferencing or phone.
- In person meetings will be held at Seattle Children's Hospital in the adolescent or sports medicine clinic, or other community locations as convenient for subject families.
- The study biostatistician and third investigator will be located at University of Washington.

21.0 Resources Available

21.1 Staff qualifications:

PIs include Drs. Rivara, Zatzick, & McCarty:

- **Dr. Rivara** is Professor & Vice Chair for Academic Affairs in the Department of Pediatrics, University of Washington School of Medicine, & Adjunct Professor in the Department of Epidemiology, School of Public Health. He is a member of the Center for Child Health Behavior & Development, Seattle Children's Research Institute. He has been involved in numerous studies including intervention trials.
- **Dr. McCarty** is a Research Professor, Department of Pediatrics, and Adjunct Research Professor, Department of Psychology, University of Washington. She is a member of the Center for Child Health Behavior and Development, of the SCRI, and is Research Director for the Division of Adolescent Medicine, Department of Pediatrics. Dr. McCarty is a licensed clinical psychologist in WA. She has extensive experience in conducting randomized trials of interventions with adolescents.
- **Dr. Zatzick** is currently Professor in the Department of Psychiatry and Behavioral Sciences, University of Washington School of Medicine. He is a core member of the research faculty at the Harborview Injury Prevention and Research Center (HIPRC). Dr. Zatzick was chairperson of the National Institute of Mental Health (NIMH) Services in Non-specialty Settings Study Section from 2009-2012 and is a standing member of the congressionally mandated Institute of Medicine four-year ongoing assessment of PTSD treatment. Dr. Zatzick was also the medical director of the Harborview Level I Trauma Center Psychiatry Consultation Liaison Service. As co-PI, Dr. Zatzick will jointly oversee all scientific aspects of the proposed R01 investigation. He will be responsible training of the MSW case managers, coaching the collaborative care team and consulting with Dr. Hilt on psychopharmacological treatment in the stepped up care interventions. He will also be responsible for assessing youth with reported suicidal ideation. He will also oversee Dr. Wang and the IT Research Consultant. Dr. McCarty will jointly with Drs. Rivara and Zatzick oversee all scientific aspects of the proposed R01 investigation. She will be responsible for overseeing the intervention delivery, fidelity to the intervention, and providing supervision to the intervention social workers.
- **Co-Investigators:**
- **Dr. Hilt.** Dr. Hilt is an Associate Professor in the Department of Psychiatry and Behavioral Sciences at the University of Washington. He has extensive experience in treating concussive sports injuries as a practicing pediatrician, a pediatric ED physician, and as a past director of psychiatric emergency services, and associate director of the child and adolescent consultation liaison psychiatry service at Seattle Children's Hospital. He is the Principal Investigator of a HRSA/MCHB-funded national, longitudinal survey that assesses experiences of children using psychotropic medication. Dr. Hilt will serve as the expert child psychopharmacologic consultant on the proposal. He will advise the stepped collaborative care intervention team on

psychotropic medication management for concussed sports injured adolescents.

- **Dr. Thomas Jinguji.** Dr. Thomas Jinguji is a clinical associate professor in the Department of Orthopedics and Sports Medicine and the Department of Pediatrics, University of Washington School of Medicine. He completed his pediatric residency and internship at the University of Washington School of Medicine. His fellowship in primary care sports medicine was also completed through the University of Washington School of Medicine. His past research in concussion includes obtaining baseline values for concussion testing (SCAT-2) and the effect of state concussion laws on concussion reporting. His current concussion work includes a review of diffusion tensor imaging in concussion and collaboration in a study involving cognitive behavioral therapy in youth athletes with prolonged concussion. He is a member of the American Academy of Pediatrics and the American Medical Society for Sports Medicine.
- **Dr. Celeste Quitiquit.** Dr. Celeste Quitiquit is a board-certified pediatrician and sports medicine doctor. She clinically works in Seattle Children's South Clinic seeing children and adolescents with any activity- or sports-related health issues and non-operative musculoskeletal conditions, and is an active clinical member of the Seattle Children's Sports Concussion Program. She has collaborated on previous concussion research around concussion reporting behavior and the association of mental health and concussion. She has experience covering collegiate and high school teams and currently volunteers her time covering local high schools assisting with sideline coverage.
- **Dr. Marisa Osorio.** Dr. Marisa Osorio is an assistant professor of rehabilitation medicine at the University of Washington. She is board-certified in pediatric rehabilitation medicine and physical medicine & rehabilitation. She has collaborated on previous concussion research involving cognitive behavioral therapy in youth athletes with prolonged concussion.
- **Study Coordinator** will have prior experience with pediatric clinical research, to include IRB, recruitment, consent, data collection, and coordinating day-to-day operations of the study. The coordinator will be responsible for all IRB preparation and submission, web-based data collection, subject recruitment procedure trainings, and research team collaboration. This includes coordinating data acquisition, management and reporting, scheduling, and coordinating interdisciplinary research and clinical conferences with the team. The research coordinator will also randomize subjects.
- **Care Managers** will have prior training or experience with care management. They will be the primary study interventionists. In the start-up period of the award, the care managers will receive study-specific training in care management, motivational interviewing, and in the cognitive behavioral

therapy intervention components by Drs. McCarty and Zatzick. The care managers will attend educational sessions that will review research and clinical information on post-injury youth behavioral and emotional disturbances, and shared patient-provider decision making. The care managers will be the team members responsible for contacting subjects randomized to the intervention group. The care managers will be responsible for documenting all of their intervention subject intervention information in the clinical registry tool. The care managers will also be the primary study interventionists charged with linking intervention subjects from non-medical referrals to acute care to primary care settings, as needed. The care manager will attend weekly meetings with the PIs.

- **Research Assistants** have prior experience with recruiting and consenting pediatric subjects. They will be supervised by the study coordinator and will assist in the recruitment, eligibility screening, consent, tracking, and follow-up of adolescents and parents. The research assistants will be blinded to subject intervention and control group status. The research assistants will be responsible for following subjects over the course of the study and for conducting follow-up assessments. The research assistants will also work to design figures, tables, and text, for final report and manuscript submissions. The research assistants will be directly supervised in all activities by the research coordinator.
- **Biostatistician** will have experience in designing, selecting measures for, and analyzing outcomes of collaborative care interventions for youth and adults suffering from anxiety and depression comorbid with other general medical conditions. The principal investigators and data analyst will have weekly meetings in which they will discuss issues relating to the randomized clinical trial study design and analysis plan.

22.0 Prior Approvals – N/A

23.0 Recruitment Methods

Subjects will be recruited from primary care medical pediatric clinics, sports medicine specialty clinics, pediatric neurology clinics, and rehabilitation medicine clinics throughout the Seattle and Western Washington region. Potential subjects will also be recruited through information posted online to include study flyers, and study advertisements

There will be active recruitment by weekly scanning of the appointment lists for the Sports Medicine, Neurology, Neurosurgery and Rehabilitation Medicine clinics at Seattle Children's Hospital main campus and four satellite locations, and the Sports Medicine and Rehabilitation Medicine clinics at Harborview Medical Center. Providers seeing potentially eligible subjects at Seattle Children's or the Sports Medicine and Rehabilitation Medicine clinics at Harborview will be contacted by study team prior to patient visits and reminded to provide the patient with study information. The research team will follow-up with interested individuals via phone, letter, or short text message

with the goal of arranging a time to talk by phone or in-person to describe the study, complete an eligibility screen, and obtain informed consent.

Recruitment materials (flyers & brochures) describing the study will also be distributed to school nurses, athletic trainers, and pediatric clinics throughout the Puget Sound to share with patients. The research team will have weekly contact with providers at these locations to learn about patients that have expressed interest in the study. The research team will follow-up with interested individuals via phone, letter, or short text message with the goal of arranging a time to talk by phone or in-person to describe the study, complete an eligibility screen, and obtain informed consent.

Recruitment materials will also be posted at community locations across the Puget Sound area, on local community internet message boards, and in waiting rooms within the offices of local medical providers. A shorter recruitment message will be used to reach potential participants via social media platforms such as Facebook and Instagram. All social media messaging and infrastructure to be used for recruitment will be submitted to the IRB for review and approval via future modification. All recruitment materials will direct parents who are interested in potentially participating to contact the study staff for more information, The research team will follow-up with individuals that express interest generated by recruitment materials via phone, letter, or short text message with the goal of arranging a time to describe the study, complete an eligibility screen, and obtain informed consent.

The research team will limit their initial recruitment contact to no more than 8 attempts per participant (4 text messages, 3 phone calls, 1 letter).

Materials to be used to recruit subjects will include:

- Study Flyer
- Parent FAQ list
- Online Ad (to be submitted to IRB in future modification)
- Study Website (to be submitted to IRB in future modification)
- Parent Approach Text message
- Parent Approach Phone script
- Parent Approach letter
- Eligibility screen

Adolescents and parents will be reimbursed for the time required to complete study recruitment and follow-up procedures. Sports injured adolescents and their parents represent a challenging population to engage and follow-up with. It is anticipated that the initial baseline assessment will require 30-45 minutes of each adolescent and parent. Adolescents and parents will receive $40 and $20 respectively for this assessment. Follow-up assessments will require between 30-60 minutes of each participant’s time, and each adolescent and parent will receive $20 for completion of the 3-month and 6-month assessments and $40 for adolescents and $20 for parents for the completion of the 12-month follow-up assessment. These amounts are consistent with reimbursements from the previous investigations by the study team. All participants will receive compensation via ClinCard.

24.0 Use of Social Media

24.1 We will place targeted study advertisement on social media platforms, like Facebook and Instagram. Individuals that click on the ad will be taken to a study webpage where they can learn more about the study and opt to be contacted by research team to learn more about the study and determine eligibility. A diverse population can be found on social media. Placing an ad on social media provides an avenue for potential subjects to learn about the study outside of the clinical setting and read through information about the study at their own pace.

24.2 Clicking on the study ad will not provide any additional information about the potential subject than is already available if they click on a different ad on social media. The study team will not have access to any personally identifiable information from potential subjects until they click through the ad, arrive at the study webpage, and choose to provide us with their information.

24.3 Ad content and study website content will be considered IRB-reviewable subject matter. All social media messaging and infrastructure to be used for recruitment will be submitted to the IRB for review and approval via future modification.

24.4 No user-generated content on public websites will be active.

25.0 Local Number of Subjects

25.1 We will recruit and consent 200 subjects locally. One hundred will be randomized to the intervention group and one hundred will be randomized to the control group.

25.2 We estimate we will need to screen 600 individuals in order to enroll 200 subjects.

26.0 Provisions to Protect the Privacy Interests of Subjects

26.1 All research activities will take place with subjects via phone, HIPAA compliant video conferencing, online survey, or in-person in a private room. Subjects can choose where they opt to take a call to complete a survey by phone, open an online survey, or engage with the care manager by phone or video conference.

26.2 Subjects will be reminded throughout their participation that they do not need to complete to any questions or research procedure that makes them uncomfortable.

26.3 Data will be contained on a secure server at the Seattle Children’s Research Institute or University of Washington when REDCap is used. Study staff will have access to this data as needed for patient contact purposes. Data analysts will be provided de-identified data. School records (grades and attendance) will be requested from schools. This information

will be entered into the REDCap database and paper copies of the school records will be kept in locked file cabinet.

27.0 Compensation for Research-Related Injury

27.1 This is a minimal risk study.

28.0 Economic Burden to Subjects

28.1 We will incentivize subjects for participation in the study. We will provide compensation for parking, as needed for in-person consent or CBT sessions.

29.0 Consent Process

29.1 We will obtain consent and assent for this research.

- Each eligible adolescent and parent (or legal guardian) will be called by a research assistant or study coordinator to obtain informed consent or schedule an in-person meeting at a time convenient for the parent (or legal guardian) and adolescent. If the adolescent and parent wish to do consent by phone, they will be emailed a copy of the consent forms to reference and take notes on during the phone call. During the consent call/meeting, the research assistant or study coordinator will first obtain consent from the parent (or legal guardian) after which they will obtain assent from the adolescent, unless the adolescent is 18 years of age in which case they will provide their own consent. Parents (or legal guardians) and 13-18 year old adolescents will sign consent/assent/parent permission forms (identical form is used to obtain all three) and adolescents 11-12 years old will sign assent forms (designed with age-appropriate language for younger adolescents). Because we are also asking parents to provide information about themselves, parents will also be asked to sign a parent participant form. Given that this is a minimal risk study, parental permission from one parent (or legal guardian) will be considered adequate. Youth who turn 18 while in the study will be re-consented either over the phone or in person either during study visits (if they are randomly assigned to the intervention group) or prior to the next data collection point following their 18th birthday. Because the intervention does not involve delivering medical care or diagnosis consent can be obtained by non-health care professional.
- The research assistant or study coordinator will explain that participation in the study is voluntary and that it's the families' choice if they want to participate. Then the research assistant or study coordinator will explain the research in lay language describing the potential harms and benefits of the study, ensuring that all topics in the consent form are explained in sufficient detail. After the explanation of the research, potential participants will be given the option to be alone with the information for as long as they need in order to feel comfortable asking questions and processing the option of participating. When they are ready to discuss the study, they will be prompted by the researcher asking, 'What questions do you have?" Assessment of the potential participant's understanding will be achieved by asking the potential participant to repeat what she/he thinks the goals or the

study are and what would occur if they chose to take part in his or her own language. These questions will be asked in a non-judgmental way, i.e. no "quizzing" of the potential participant and ensuring that they remain comfortable. If the participant shows through their review of the study that more explanation is needed, the research coordinator or research assistant will make sure to cover these points a second time, still ensuring that the participant does not feel minimized in the discussion. As needed, the consent meeting will be re-scheduled if the family requests more time to make their decision. For adolescents and parents choosing to consent by phone, the research assistant or study coordinator will provide a brief review of the consent documents during the in-person baseline visits and then have the adolescent and parent sign the appropriate consent documents.

- At the time of consent, participants will be allowed to opt-in to the sharing of their study data with the Federal Interagency Traumatic Brain Injury Research (FITBIR) Informatics System, a central repository and resource for sharing data that was developed by the Department of Defense (DOD) and the National Institutes of Health (NIH) to promote collaboration, accelerate research, and advance knowledge on the characterization, prevention, diagnosis and treatment of traumatic brain injury (TBI). As a NICHD NIH funded study, we are required to share results from this study with the Federal Interagency Traumatic Brain Injury Research (FITBIR) Informatics System repository. All links with the participant's identity will be removed from the data before study data is shared with FITBIR. Participants will be given the opportunity to opt-out of sharing their study data with FITBIR on the consent form. When study data is uploaded to FITBIR, which will occur within one year following the end of the performance period for the NICHD Award, the study team will exclude data of any participants who have opted out from sharing their data.

- Completion of 3, 6, and 12 month surveys will be used as indicators of ongoing consent.
- We will follow "SOP: Informed Consent Process for Research (HRP-090).

Non-English Speaking Subjects

We are not enrolling any non-English speaking subjects as it would not be logistically or financially feasible, at this time, to provide translation and interpreter services during consent, all four data collection time points, and all interactions with the care manager for the participants randomized to the intervention arm of the study. Interactions with the care manager for participants randomized to the intervention arm will involve intensive communication via multiple modalities (e.g. phone, text, email, in-person, and video conferencing) during the first 6 months of participation and need to be timely and responsive to each participant.

Waiver or Alteration of Consent Process (consent/parental permission will not be obtained, required information will not be disclosed, or the research involves deception); Waiver or Alteration of HIPAA

We are requesting an alteration of HIPAA because we will not obtain written signature on an authorization of HIPAA form prior to collecting a limited set of information about potential participants to be used to determine whether or not they meet study eligibility criteria. As is desired when requesting an alteration of HIPAA, we can confirm that 1) the PHI collected during screening activities is necessary for the research, 2) use or disclosure of the PHI obtained during screening activities involves no more than a minimal risk to the privacy of individuals, based on the presence of the following: a) we have a plan to protect the identifiers from improper use and disclosure b) we will destroy identifiers at the earliest opportunity consistent with conduct of the research, unless there is a health or research justification for retaining the identifiers or such retention is otherwise required by law, c) the PHI will not be reused or disclosed to any other person or entity, except as required by law, for authorized oversight of the research study, or for other research for which the use or disclosure of protected health information for which an authorization or opportunity to agree or object is not required, d) the research cannot be practicably conducted without the alteration given the number of potential participants and that they are spread across the Puget Sound region, and e) the research cannot be practicably conducted without access to and use of the PHI for screening purposes.

Subjects who are not yet adults (infants, children, teenagers)

- Participant age will be confirmed during consent meeting by asking parent for youth's date of birth. Youth younger than age 18 will not yet be considered adults..

30.0 Process to Document Consent in Writing

30.1 We will obtain documentation of consent in writing during consent conferences following SOP: Written Documentation of Consent (HRP-091).

31.0 Drugs or Devices N/A

32.0 Good Clinical Practice N/A

1. *Barlow KM, Crawford S, Stevenson A, Sandhu SS, Belanger F, Dewey D. Epidemiology of postconcussion syndrome in pediatric mild traumatic brain injury. Pediatrics. Aug 2010;126(2):e374-381.*

2. *Chrisman SP, Richardson LP. Prevalence of Diagnosed Depression in Adolescents With History of Concussion. The Journal of adolescent health : official publication of the Society for Adolescent Medicine. Dec 3 2013.*

3. *Hajek CA, Yeates KO, Gerry Taylor H, et al. Relationships among post-concussive symptoms and symptoms of PTSD in children following mild traumatic brain injury. Brain injury. Feb 2010;24(2):100-109.*

4. *Vargas G, Rabinowitz A, Meyer J, Arnett PA. Predictors and prevalence of postconcussion depression symptoms in collegiate athletes. J Athl Train. Mar 2015;50(3):250-255.*

5. *McCarty CA, Zatzick D, Stein E, et al. Collaborative Care for Adolescents With Persistent Postconcussive Symptoms: A Randomized Trial. Pediatrics. Sep 13 2016.*

6. *Langlois JA, Rutland-Brown W, Wald MM. The epidemiology and impact of traumatic brain injury: a brief overview. J Head Trauma Rehabil. Sep-Oct 2006;21(5):375-378.*

7. *Peterson RL, Kirkwood MW, Taylor HG, Stancin T, Brown TM, Wade SL. Adolescents' internalizing problems following traumatic brain injury are related to parents' psychiatric symptoms. J Head Trauma Rehabil. Sep-Oct 2013;28(5):E1-12.*

8. *Institute of Medicine (IOM) and National Research Council (NRC). Sports-related concussions in youth: Improving the science, changing the culture. Washington, DC: The National Academies Press; 2013.*

9. *Lundin A, de Boussard C, Edman G, Borg J. Symptoms and disability until 3 months after mild TBI. Brain injury. Jul 2006;20(8):799-806.*

10. *Ellis MJ, Ritchie LJ, Koltek M, et al. Psychiatric outcomes after pediatric sports-related concussion. Journal of neurosurgery. Pediatrics. Sep 11 2015:1-10.*

11. *Waljas M, Iverson GL, Lange RT, et al. A prospective biopsychosocial study of the persistent post-concussion symptoms following mild traumatic brain injury. J Neurotrauma. Apr 15 2015;32(8):534-547.*

12. *Fann JR, Hart T, Schomer KG. Treatment for depression after traumatic brain injury: A systematic review. J Neurotrauma. Dec 2009;26(12):2383-2402.*

13. *Silverberg ND, Iverson GL. Is rest after concussion "the best medicine?": recommendations for activity resumption following concussion in athletes, civilians, and military service members. J Head Trauma Rehabil. Jul-Aug 2013;28(4):250-259.*

14. *Ponsford J, Willmott C, Rothwell A, et al. Impact of early intervention on outcome after mild traumatic brain injury in children. Pediatrics. Dec 2001;108(6):1297-1303.*

15. *Giza CC, Kutcher JS, Ashwal S, et al. Summary of evidence-based guideline update: evaluation and management of concussion in sports: report of the Guideline Development Subcommittee of the American Academy of Neurology. Neurology. Jun 11 2013;80(24):2250-2257.*

16. *Kaye AJ, Gallagher R, Callahan JM, Nance ML. Mild traumatic brain injury in the pediatric population: The role of the pediatrician in routine follow-up. J Trauma. Jun 2010;68(6):1396-1400.*

17. *Gioia GA. Multimodal evaluation and management of children with concussion: using our heads and available evidence. Brain injury. 2015;29(2):195-206.*

18. *Pangilinan PH, Giacoletti-Argento A, Shellhaas R, Hurvitz EA, Hornyak JE. Neuropharmacology in pediatric brain injury: A review. Pm R. Dec 2010;2(12):1127-1140.*

19. *Wade SL, Stancin T, Kirkwood M, Brown TM, McMullen KM, Taylor HG. Counselor-Assisted Problem Solving (CAPS) Improves Behavioral Outcomes in Older Adolescents With Complicated Mild to Severe TBI. J Head Trauma Rehabil. May 1 2013.*

20. *Raj SP, Wade SL, Cassedy A, et al. Parent Psychological Functioning and Communication Predict Externalizing Behavior Problems After Pediatric Traumatic Brain Injury. J Pediatr Psychol. Sep 23 2013.*

21. *Arnett AB, Peterson RL, Kirkwood MW, et al. Behavioral and cognitive predictors of educational outcomes in pediatric traumatic brain injury. J Int Neuropsychol Soc. Sep 2013;19(8):881-889.*

22. *Fulton JB, Yeates KO, Taylor HG, Walz NC, Wade SL. Cognitive predictors of academic achievement in young children 1 year after traumatic brain injury. Neuropsychology. May 2012;26(3):314-322.*

23. *Zatzick D, Roy-Byrne P, Russo J, et al. A randomized effectiveness trial of stepped collaborative care for acutely injured trauma survivors. Arch Gen Psychiatry. May 2004;61(5):498-506.*

24. *Richardson LP, Ludman E, McCauley E, et al. Collaborative care for adolescents with depression in primary care: a randomized clinical trial. JAMA. Aug 27 2014;312(8):809-816.*

25. *Zatzick D, Russo J, Lord SP, et al. Collaborative care intervention targeting violence risk behaviors, substance use, and posttraumatic stress and depressive symptoms in injured adolescents: A randomized clinical trial. JAMA Pediatr. Jun 2014;168(6):532-539.*

26. *A Randomized Clinical Trial of a Collaborative Care Intervention for Adolescents with Persistent Post-Concussive Symptoms. 2016.*

27. *Hopkins K, Crosland P, Elliott N, Bewley S. Diagnosis and management of depression in children and young people: summary of updated NICE guidance. BMJ. 2015;350:h824.*

28. *Kennard BD, Silva SG, Tonev S, et al. Remission and recovery in the Treatment for Adolescents with Depression Study (TADS): acute and long-term outcomes. J Am Acad Child Adolesc Psychiatry. Feb 2009;48(2):186-195.*

29. *Walkup JT, Albano AM, Piacentini J, et al. Cognitive behavioral therapy, sertraline, or a combination in childhood anxiety. N Engl J Med. Dec 25 2008;359(26):2753-2766.*

30. *Spence SH, Donovan CL, March S, et al. A randomized controlled trial of online versus clinic-based CBT for adolescent anxiety. J Consult Clin Psychol. Jul 11 2011.*

31. *Bisson J, Andrew M. Psychological treatment of post-traumatic stress disorder (PTSD). Cochrane Database Syst Rev. 2007(3):CD003388.*

32. *Roberts NP, Kitchiner NJ, Kenardy J, Bisson J. Multiple session early psychological interventions for the prevention of post-traumatic stress disorder. Cochrane Database Syst Rev. 2009(3):CD006869.*

33. *Institute of Medicine (IOM). Treatment for posttraumatic stress disorder in military and veteran populations: Initial assessment. Washington, DC: The National Academies Press; 2012.*

34. *Foa EB, Keane TM, Friedman MJ, Cohen JA. Effective treatments for PTSD: Practice guidelines from the International Society for Traumatic Stress Studies. 2 ed. New York: The Guilford Press; 2009.*

35. *Wang PS, Simon GE, Avorn J, et al. Telephone screening, outreach, and care management for depressed workers and impact on clinical and work productivity outcomes: a randomized controlled trial. JAMA. Sep 26 2007;298(12):1401-1411.*

36. *Bell KR, Fann JR, Brockway JA, et al. Telephone Problem Solving for Service Members with Mild Traumatic Brain Injury: A Randomized, Clinical Trial. J Neurotrauma. Oct 13 2016.*

37. *Wade SL, Taylor HG, Cassedy A, et al. Long-term behavioral outcomes after a randomized, clinical trial of counselor-assisted problem solving for adolescents with complicated mild-to-severe traumatic brain Injury. J Neurotrauma. Jul 1 2015;32(13):967-975.*

38. *Hilt RJ, Romaire MA, McDonell MG, et al. The Partnership Access Line: evaluating a child psychiatry consult program in Washington State. JAMA pediatrics. Feb 2013;167(2):162-168.*

39. *Garber J, Clarke GN, Weersing VR, et al. Prevention of depression in at-risk adolescents: A randomized controlled trial. Jama. 2009;301(21):2215-2224.*

40. *Bridge JA, Iyengar S, Salary CB, et al. Clinical response and risk for reported suicidal ideation and suicide attempts in pediatric antidepressant treatment: a meta-analysis of randomized controlled trials. Jama. Apr 18 2007;297(15):1683-1696.*

41. *Donnelly CL. Pharmacologic treatment approaches for children and adolescents with posttraumatic stress disorder. Child Adolesc Psychiatr Clin N Am. Apr 2003;12(2):251-269.*

42. *March J, Silva S, Petrycki S, et al. Fluoxetine, cognitive-behavioral therapy, and their combination for adolescents with depression: Treatment for Adolescents With Depression Study (TADS) randomized controlled trial. Jama. Aug 18 2004;292(7):807-820.*

43. *Huemer J, Erhart F, Steiner H. Posttraumatic stress disorder in children and adolescents: a review of psychopharmacological treatment. Child psychiatry and human development. Dec 2010;41(6):624-640.*

44. *Bridge JA, Salary CB, Birmaher B, Asare AG, Brent DA. The risks and benefits of antidepressant treatment for youth depression. Ann Med. 2005;37(6):404-412.*

45. *Compton SN, Kratochvil CJ, March JS. Pharmacotherapy for anxiety disorders in children and adolescents: an evidence-based medicine review. Pediatr Ann. Sep 2007;36(9):586-590, 592, 594-588.*

46. *Zatzick D, Russo J, Lord SP, et al. A pragmatic trial of a collaborative care intervention targeting violence risk behaviors, substance use and posttraumatic stress and depressive symptoms in injured adolescents. JAMA Pediatr. In Press.*

47. *Zatzick D, Roy-Byrne P, Russo J, et al. A randomized effectiveness trial of stepped collaborative care for acutely injured trauma survivors. Arch Gen Psychiat. May 2004;61(5):498-506.*

48. *Zatzick D, Jurkovich G, Rivara FP, et al. A randomized stepped care intervention trial targeting posttraumatic stress disorder for surgically hospitalized injury survivors. Ann Surg. 2013;257(3):390-399.*

49. *Kessler RC, Sonnega A, Bromet E, Hughes M, Nelson CB. Posttraumatic stress disorder in the National Comorbidity Survey. Arch Gen Psychiatry. Dec 1995;52(12):1048-1060.*

50. *Yeates KO, Luria J, Bartkowski H, Rusin J, Martin L, Bigler ED. Postconcussive symptoms in children with mild closed head injuries. J Head Trauma Rehabil. Aug 1999;14(4):337-350.*

51. *Koepsell TD, Zatzick DF, Rivara FP. Estimating the population impact of preventive interventions from randomized trials. Am J Prev Med. Feb 2011;40(2):191-198.*

52. *Altman DG, Schulz KF, Moher D, et al. The revised CONSORT statement for reporting randomized trials: Explanation and elaboration. Ann Intern Med. 2001;134(8):663-694.*

53. *Zatzick D, Kang SM, Hinton WL, et al. Posttraumatic concerns: A patient-centered approach to outcome assessment after traumatic physical injury. Med Care. 2001;39(4):327-339.*

54. *Zatzick D, Russo J, Rajotte E, et al. Strengthening the patient-provider relationship in the aftermath of physical trauma through an understanding of the nature and severity of posttraumatic concerns. Psychiatry. 2007;70(3):260-273.*

55. *Geiss Trusz S, Wagner A, Russo J, Love J, Zatzick D. Assessing barriers to care and readiness for cognitive behavioral therapy in early acute care PTSD interventions. Psychiatry. 2011;74(3):207-223.*

56. *Zatzick D, O'Connor SS, Russo J, et al. Technological innovation targeting efficient intervention for PTSD and comorbidity after injury: A randomized clinical trial In preparation for submission.*

57. *Russo J, Katon W, Zatzick D. The development of a population-based automated screening procedure for PTSD in acutely injured hospitalized trauma survivors. Gen Hosp Psychiatry. Sep-Oct 2013;35(5):485-491.*

58. *Ursano RJ, Bell C, Eth S, et al. Practice guideline for the treatment of patients with acute stress disorder and posttraumatic stress disorder. Arlington, VA: American Psychiatric Association;2004.*

59. *Brent D, Emslie G, Clarke G, et al. Switching to another SSRI or to venlafaxine with or without cognitive behavioral therapy for adolescents with SSRI-resistant depression: the TORDIA randomized controlled trial. Jama. Feb 27 2008;299(8):901-913.*

60. *Raskind MA, Peskind ER, Hoff DJ, et al. A parallel group placebo controlled study of prazosin for trauma nightmares and sleep disturbance in combat veterans with post-traumatic stress disorder. Biol Psychiatry. 2007;61(8):928-934.*

61. *Zatzick D, Russo J, Lord SP, et al. A Pragmatic Trial of a Collaborative Care Intervention Targeting Violence Risk Behaviors, Substance Use and Posttraumatic Stress and Depressive Symptoms in Injured Adolescents. Jama Pediatrics. 2014;In press.*

62. *Ayr LK, Yeates KO, Taylor HG, Browne M. Dimensions of postconcussive symptoms in children with mild traumatic brain injuries. J Int Neuropsychol Soc. Jan 2009;15(1):19-30.*

63. *Richardson LP, McCauley E, Grossman DC, et al. Evaluation of the patient health questionnaire-9 item for detecting major depression among adolescents. Pediatrics. 2010;126(6):1117-1123.*

64. *Lowe B, Decker O, Muller S, Brahler E, Schelberg D. Validation and standardization of the General Anxiety disorder Screener (GAD-7) in the general population. Med care. 2008;46:266-274.*

65. *Mathyssek CM, Olino TM, Hartman CA, Ormel J, Verhulst FC, Van Oort FV. Does the Revised Child Anxiety and Depression Scale (RCADS) measure anxiety symptoms consistently across adolescence? The TRAILS study. Int J Methods Psychiatr Res. Mar 2013;22(1):27-35.*

66. *PROMIS. Dynamic Tools to Measure Health Outcomes from the Patient Perspective. http://www.nihpromis.org.*

67. *Cella D, Riley W, Stone A, et al. The Patient-Reported Outcomes Measurement Information System (PROMIS) developed and tested its first wave of adult self-reported health outcome item banks: 2005-2008. J Clin Epidemiol. Nov 2010;63(11):1179-1194.*

68. *National Institute of Health. NIH Toolbox for the Assessment of Neurological and Behavioral Function (NIH Toolbox). Available at: http://www.nihtoolbox.org/WhatAndWhy/Pages/default.aspx.*

69. *von Baeyer CL. Children's self-reports of pain intensity: scale selection, limitations and interpretation. Pain research & management : the journal of the Canadian Pain Society = journal de la societe canadienne pour le traitement de la douleur. Autumn 2006;11(3):157-162.*

70. *Attkisson CC, Zwick R. The client satisfaction questionnaire. Psychometric properties and correlations with service utilization and psychotherapy outcome. Eval Program Plann. 1982;5(3):233-237.*

71. *McLaughlin KA, Koenen KC, Hill ED, et al. Trauma exposure and posttraumatic stress disorder in a national sample of adolescents. Journal of the American Academy of Child and Adolescent Psychiatry. Aug 2013;52(8):815-830 e814.*

72. *Zatzick D, Jurkovich G, Rivara FP, et al. A randomized stepped care intervention trial targeting posttraumatic stress disorder for surgically hospitalized injury survivors. Ann Surg. Mar 2013;257(3):390-399.*

73. *Zatzick DF, Rivara FP, Jurkovich GJ, et al. Multisite investigation of traumatic brain injuries, posttraumatic stress disorder, and self-reported health and cognitive impairments. Arch Gen Psychiatry. Dec 2010;67(12):1291-1300.*

74. *MacKenzie EJ, Rivara FP, Jurkovich GJ, et al. A national evaluation of the effect of trauma-center care on mortality. N Engl J Med. Jan 26 2006;354(4):366-378.*

75. *Kroenke K, Spitzer RL, Williams JB. The PHQ-9: Validity of a brief depression severity measure. J Gen Intern Med. Sep 2001;16(9):606-613.*

76. *Gibbons RD, Hedeker D. Application of random-effects probit regression models. J Consult Clin Psychol. 1994;62(2):285-296.*

77. *Gibbons RD, Hedeker D, DuToit S. Advances in analysis of longitudinal data. Annu Rev Clin Psychol. 2010;6:79-107.*

78. *Gibbons RD, Hedeker D, Elkin I, et al. Some conceptual and statistical issues in analysis of longitudinal psychiatric data. Arch Gen Psychiat. 1993;50:739-750.*

79. *McCulloch CE, Searle SR. Generalized, linear, and mixed models. Wiley; 2000.*

80. *King DW, King LA, Bachrach PS, McArdele JJ. Contemporary approaches to missing data: The glass is really half full. PTSD Research Quarterly. 2001;12:1-6.*

81. *Siddique J, Brown CH, Hedeker D, et al. Missing data in longitudinal trials - part B, analytic issues. Psychiatric Annals. 2008;38(12):793-801.*

82. *National Research Council. The Prevention and Treatment of Missing Data in Clinical Trials. In: Panel on Handling Missing Data in Clinical Trials. Committee on National Statistics DoBaSSaE, ed. Washington, DC: The National Academies Press; 2010:1-163.*

83. *Little RJ, Rubin DB. Statistical analysis with missing data. 2nd ed. Hoboken, NJ: Wiley; 2002.*

84. *Hintze JL. PASS User's Guide. Kaysville: NCSS; 2000.*

85. *Cohen J. Statistical power analysis for the behavioral sciences. 2nd ed. Hillsdale, NJ: Lawrence Earlbaum Associates; 1988.*

86. *Browner WS, Black D, Newman TB, Hulley SB. Estimating sample size and power. In: Hulley SB, Cummings SR, eds. Designing clinical research: An epidemiologic approach. Baltimore, MD: Williams and Wilkins; 1988.*

87. *Yeates KO. Mild traumatic brain injury and postconcussive symptoms in children and adolescents. J Int Neuropsychol Soc. Nov 2010;16(6):953-960.*

88. *Kraemer HC, Mintz J, Noda A, Tinklenberg J, Yesavage JA. Caution regarding the use of pilot studies to guide power calculations for study proposals. Archives of general psychiatry. May 2006;63(5):484-489.*

89. *Leon AC, Davis LL, Kraemer HC. The role and interpretation of pilot studies in clinical research. J Psychiatr Res. May 2011;45(5):626-629.*

90. *Zatzick DF, Grossman DC, Russo J, et al. Predicting posttraumatic stress symptoms longitudinally in a representative sample of hospitalized injured adolescents. J Am Acad Child Adolesc Psychiatry. October 2006;45(10):1188-1195.*

91. *Zatzick D, Jurkovich G, Fan MY, et al. The association between posttraumatic stress and depressive symptoms, and functional outcomes in adolescents followed longitudinally after injury hospitalization. Archives of Pediatrics and Adolescent Medicine. 2008;162(7):642-648.*

92. *Zatzick D, Jurkovich G, Fan MY, et al. Posttraumatic stress and depressive symptoms as predictors of adolescent functional impairment after physical injury. Archives of Pediatrics and Adolescent Medicine. 2007;in press.*

93. *Zatzick DF, Jurkovich G, Wang J, Rivara FP. Variability in the characteristics and quality of care for injured youth treated at trauma centers. J Pediatr. Dec 2011;159(6):1012-1016.*

94. *Zatzick D, Russo J, Grossman D, et al. Posttraumatic stress and depressive symptoms, alcohol use, and recurrent traumatic life events in a representative sample of hospitalized injured adolescents and their parents. J Pediatr Psychol. 2006;31(4):377-378.*

95. *Kernic MA, Holt VL, Wolf ME, McKnight B, Huebner CE, Rivara FP. Academic and school health issues among children exposed to maternal intimate partner abuse. Archives of Pediatrics and Adolescent Medicine. 2002;156(6):549-555.*

96. *Rivara FP, Calonge N, Thompson RS. Population-based study of unintentional injury incidence and impact during childhood. Am J Public Health. 1989;79(8):990-994.*

97. *McCarty CA, Russo J, Grossman DC, et al. Adolescents with suicidal ideation: Health care use and functioning. Acad Pediatr. Sep-Oct 2011;11(5):422-426.*

98. *Richardson LP, McCauley E, McCarty CA, et al. Predictors of persistence after a positive depression screen among adolescents. Pediatrics. Dec 2012;130(6):e1541-1548.*

99. *Katon W, Richardson L, Russo J, et al. Depressive symptoms in adolescence: the association with multiple health risk behaviors. Gen Hosp Psychiatry. May-Jun 2010;32(3):233-239.*

100. *Richardson LP, Rockhill C, Russo JE, et al. Evaluation of the PHQ-2 as a brief screen for detecting major depression among adolescents. Pediatrics. May 2010;125(5):e1097-1103.*

101. *Zatzick DF, Grossman DC. Association between traumatic injury and psychiatric disorders and medication prescription to youths aged 10-19. Psychiatr Serv. Mar 2011;62(3):264-271.*
