# Supplementary material for: Effect of Collaborative Care on Persistent Postconcussive Symptoms in Adolescents: A Randomized Clinical Trial
Source: JAMA Netw Open. 2021 Feb 26;4(2):e210207. doi: 10.1001/jamanetworkopen.2021.0207 (PMC7910815; doi:10.1001/jamanetworkopen.2021.0207)
Supplement: Supplement 2. — eTable 1. Proportion of Adolescents Using Healthcare Services in the Collaborative Care and Usual Care Groups Across Time eTable 2. Proportion of Adolescents Receiving SSRIs and Tricyclic Antidepressants in the Collaborative Care and Usual Care Groups Across Time eTable 3. Health Related Quality of Life Subscales in the Collaborative Care and Usual Care Groups Across Time, Mean (SD) eTable 4. Sleep Quality Differences in the Collaborative Care and Usual Care Groups Across Time, Mean (SD) eFigure 1. Post-Concussive Symptoms (HBI) in the Collaborative Care and Usual Care Groups by Parent Report eFigure 2. Health-Related Quality of Life (Peds-QL) in the Collaborative Care and Usual Care Groups by Parent Report eFigure 3. Proportion of Adolescents Indicating Suicidal Ideation in the Collaborative Care and Usual Care Groups Across Time [file jamanetwopen-e210207-s002.pdf]

Supplemental Online Content

McCarty CA, Zatzick DF, Marcynyszyn LA, et al. Effect of collaborative care on persistent postconcussive symptoms in adolescents: a randomized clinical trial. *JAMA Netw Open*. 2021;4(2):e210207. doi:10.1001/jamanetworkopen.2021.0207

eTable 1. Proportion of Adolescents Using Healthcare Services in the Collaborative Care and Usual Care Groups Across Time
eTable 2. Proportion of Adolescents Receving SSRIs and Tricyclic Antidepressants in the Collaborative Care and Usual Care Groups Across Time
eTable 3. Health Related Quality of Life Subscales in the Collaborative Care and Usual Care Groups Across Time, Mean (SD)
eTable 4. Sleep Quality Differences in the Collaborative Care and Usual Care Groups Across Time, Mean (SD)
eFigure 1. Post-Concussive Symptoms (HBI) in the Collaborative Care and Usual Care Groups by Parent Report
eFigure 2. Health-Related Quality of Life (Peds-QL) in the Collaborative Care and Usual Care Groups by Parent Report
eFigure 3. Proportion of Adolescents Indicating Suicidal Ideation in the Collaborative Care and Usual Care Groups Across Time

This supplemental material has been provided by the authors to give readers additional information about their work.

© 2021 McCarty CA et al. *JAMA Network Open.*

eTable 1. Proportion of Adolescents Using Healthcare Services in the Collaborative Care and Usual Care Groups Across Time[a]

	Study enrollment to 3 months		3 to 6 months		6 to 12 months	
	UC (n=98)	CC (n=99)	UC (n=96)	CC (n=98)	UC (n=93[a])	CC (n=98[a])
Mental health care	40.8	47.5	43.8[b]	58.2[b]	45.2	48.0
Primary care	66.3	62.6	68.8	55.1	72.0	69.4
Sports medicine	55.1	55.6	42.7	40.8	29.0	30.6
Rehabilitation medicine	28.6	31.2	18.8	14.3	11.8	9.2
Other healthcare visit	37.8	37.4	33.3	30.6	24.7	34.7

Abbreviations: CC, Collaborative Care; UC, Usual Care.
Group by time interactions adjusted for gender, age, and time elapsed since the concussion injury event.
Healthcare services are based on parent report.
[a] One parent from each group did not complete the healthcare utilization survey at 12 months.
[b] Adjusted risk ratio for CC vs. UC mental health care differences at 3 to 6 months relative to baseline = 1.80 (95% CI: 1.13 to 2.85).

© 2021 McCarty CA et al. *JAMA Network Open.*

eTable 2. Proportion of Adolescents Receving SSRIs and Tricyclic Antidepressants in the Collaborative Care and Usual Care Groups Across Time

	3 months prior to study enrollment		Study enrollment to 3 months		3 to 6 months		6 to 12 months	
	UC (n=99)	CC (n=101)	UC (n=98)	CC (n=99)	UC (n=96)	CC (n=98)	UC (n=93[a])	CC (n=98[a])
SSRIs	7.1	7.9	13.3	16.2	18.8	17.4	22.3	20.2
Tricyclic antidepressants	3.0	2.0	7.1	5.1	6.3	2.0	2.1	4.0

Abbreviations: CC, Collaborative Care; SSRIs, selective serotonin reuptake inhibitors; UC, Usual Care.
Medication use is based on parent report.
All differences are nonsignificant ($P > 0.05$).
[a] One parent from each group did not complete the survey about medication use at 12 months.

© 2021 McCarty CA et al. *JAMA Network Open.*

eTable 3. Health Related Quality of Life Subscales in the Collaborative Care and Usual Care Groups Across Time, Mean (SD)

	Baseline		3 month		6 month		12 month	
	UC (n=99)	CC (n=101)	UC (n=98)	CC (n=98)	UC (n=95)	CC (n=99)	UC (n=95)	CC (n=99)
Physical	66.7(19.4)	68.1(19.8)	82.9(15.3)	83.3(18.6)	84.9(14.9)	85.5(19.3)	84.9(15.8)	88.5(16.4)
Emotional	66.8(20.3)	67.7(21.3)	73.2(22.3)	78.1(21.1)	74.4(22.4)[a]	81.4(20.9)[a]	71.9(24.5)[a]	81.4(21.4)[a]
Social	85.1(14.9)	81.8(18.4)	86.4(15.6)	85.1(17.6)	88.7(14.3)	88.2(16.6)	88.7(15.7)[a]	90.2(15.7)[a]
School	47.7(24.2)	48.5(22.0)	66.2(22.8)	68.5(26.6)	70.1(22.4)	74.3(23.4)	69.2(20.7)	73.4(24.2)
Total score	66.6(15.2)	66.5(15.5)	77.2(15.2)	78.7(17.9)	79.5(15.5)	82.3(17.4)	78.7(16.1)[a]	83.4(16.6)[a]

Abbreviations: CC, Collaborative Care; UC, Usual Care.
All subscales are from the Pediatric Quality of Life Inventory, based on adolescent report.
Subscales are scored from 0-100, with higher scores indicating better quality of life.
[a] Differences from baseline between groups are statistically significant ($P < 0.05$).

© 2021 McCarty CA et al. *JAMA Network Open.*

eTable 4. Sleep Quality Differences in the Collaborative Care and Usual Care Groups Across Time, Mean (SD)

	**Baseline**		**3 month**		**6 month**		**12 month**	
	UC (n=99)	CC (n=101)	UC (n=98)	CC (n=98)	UC (n=95)	CC (n=99)	UC (n=95)	CC (n=99)
Going to bed	3.9(1.2)	3.8(1.0)	3.7(1.1)[a]	4.1(1.1)[a]	3.9(1.2)	4.0(1.1)	3.8(1.2)[a]	4.2(1.1)[a]
Falling asleep/reinitiating sleep	4.5(1.1)	4.3(1.0)	4.5(1.1)[a]	4.7(1.1)[a]	4.6(1.2)	4.7(1.1)	4.6(1.1)[a]	4.8(1.2)[a]
Return to wakefulness	2.4(1.1)	2.5(1.1)	2.6(1.2)[a]	3.1(1.3)[a]	2.7(1.2)	3.0(1.3)	2.7(1.3)[a]	3.2(1.4)[a]
Overall total	3.9(0.9)	3.8(0.8)	3.9(0.8)[a]	4.2(0.9)[a]	4.0(1.0)[a]	4.2(0.9)[a]	4.0(0.9)[a]	4.3(0.9)[a]

Abbreviations: CC, Collaborative Care; UC, Usual Care.
All scales are from the Adolescent Sleep-Wake Scale—Short Form, based on adolescent report.
Scales are scored on a 1-6 Likert scale, with higher scores indicating better sleep quality.
[a] Differences from baseline between groups are statistically significant ($P < 0.05$).

© 2021 McCarty CA et al. *JAMA Network Open.*

eFigure 1. Post-Concussive Symptoms (HBI) in the Collaborative Care and Usual Care Groups by Parent Report

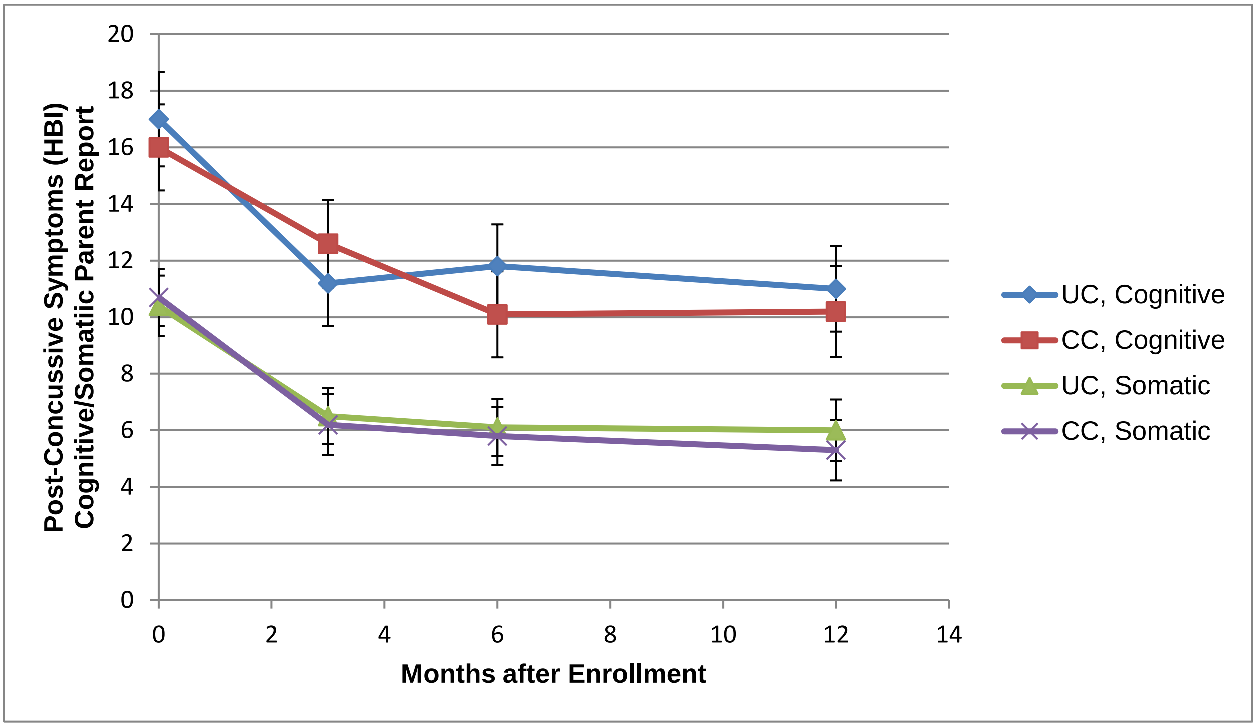

Abbreviations: CC, collaborative care; HBI, Health Behavior Inventory; UC, usual care.
Means adjusted for gender, age, and time elapsed since the concussion injury event.

© 2021 McCarty CA et al. *JAMA Network Open.*

eFigure 2. Health-Related Quality of Life (Peds-QL) in the Collaborative Care and Usual Care Groups by Parent Report

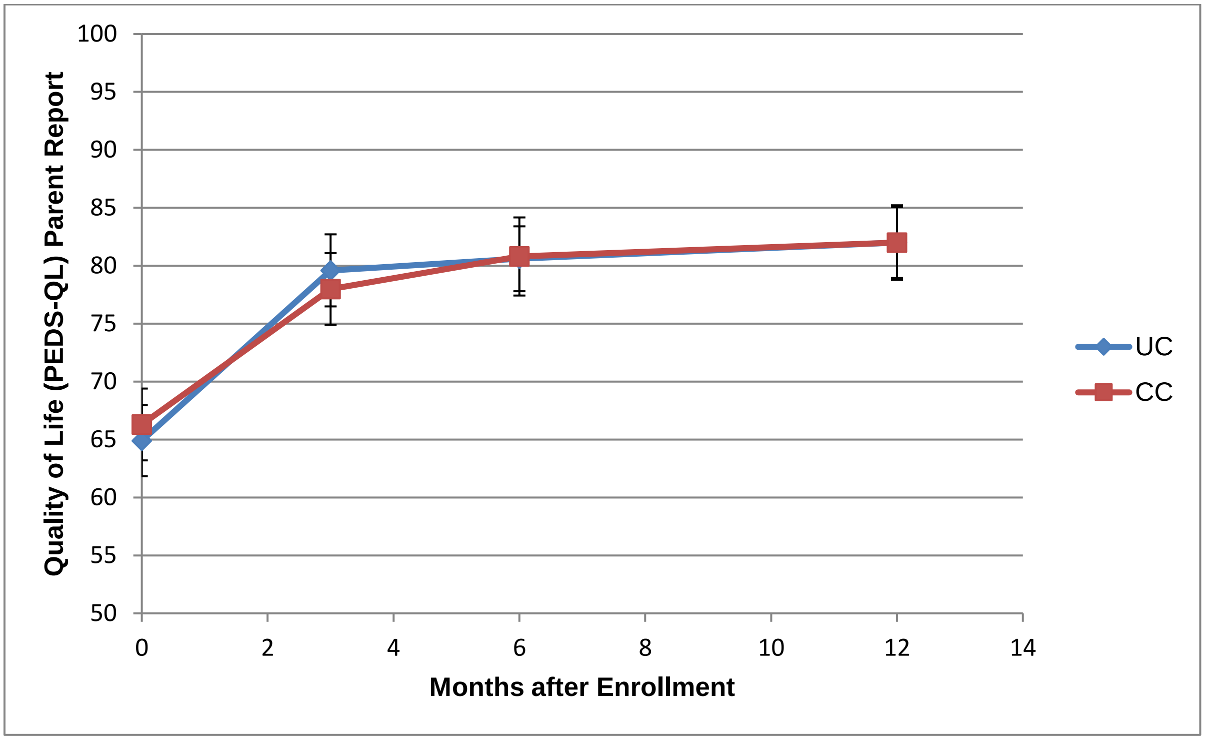

Abbreviation: CC, collaborative care; PedsQL, Pediatric Quality of Life Inventory; UC, usual care.
Means adjusted for gender, age, and time elapsed since the concussion injury event.

© 2021 McCarty CA et al. *JAMA Network Open.*

eFigure 3. Proportion of Adolescents Indicating Suicidal Ideation in the Collaborative Care and Usual Care Groups Across Time[a]

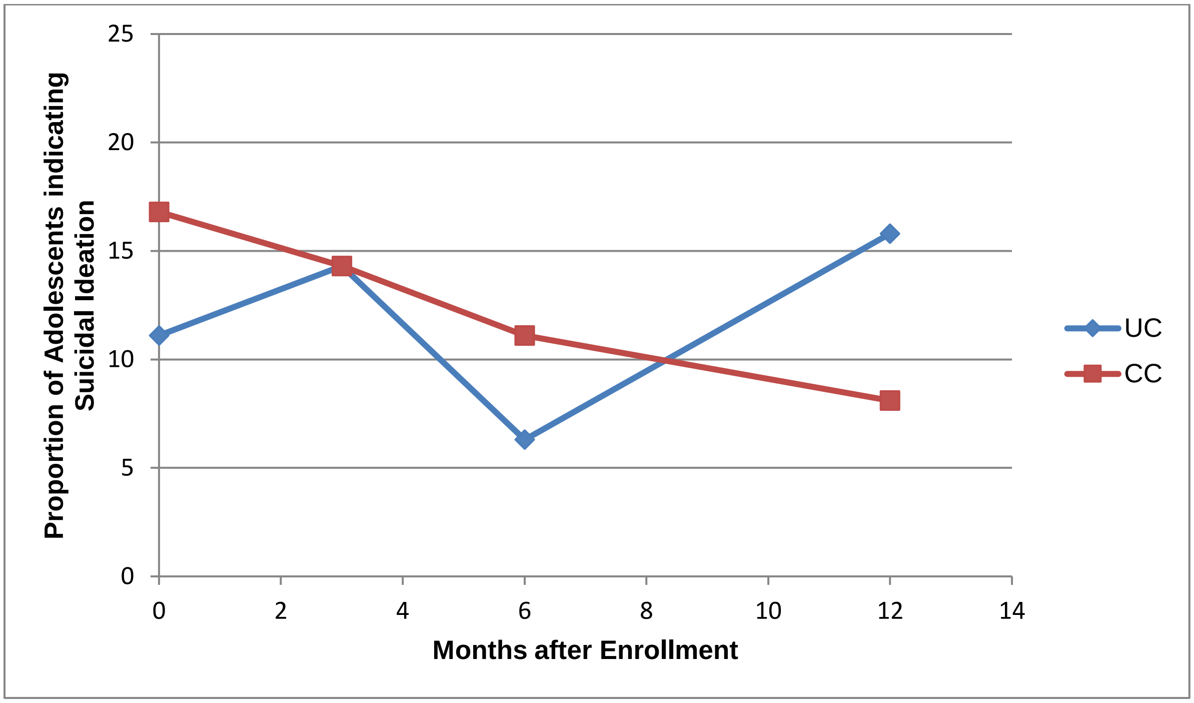

Abbreviations: CC, Collaborative Care; UC, Usual Care.
Patient Health Questionnaire-9 scores ($\geq$1) on the 9th item were used as an indicator of suicidal ideation.
[a] Proportions presented are unadjusted.

© 2021 McCarty CA et al. *JAMA Network Open.*
