# Supplementary material for: Effect of Collaborative Care on Persistent Postconcussive Symptoms in Adolescents: A Randomized Clinical Trial
Source: JAMA Netw Open. 2021 Feb 26;4(2):e210207. doi: 10.1001/jamanetworkopen.2021.0207 (PMC7910815; doi:10.1001/jamanetworkopen.2021.0207)
Supplement: Supplement 3. — Data Sharing Statement [file jamanetwopen-e210207-s003.pdf]

Data Sharing Statement

McCarty. Effect of Collaborative Care on Persistent Postconcussive Symptoms in Adolescents. *JAMA Netw Open*. Published February 26, 2021. doi:10.1001/jamanetworkopen.2021.0207

Data
Data available: No
